# Assumptions of Mixed Treatment Comparisons in Health Technology Assessments - Challenges and Possible Steps for Practical Application

**DOI:** 10.1371/journal.pone.0160712

**Published:** 2016-08-10

**Authors:** Stefanie Reken, Sibylle Sturtz, Corinna Kiefer, Yvonne-Beatrice Böhler, Beate Wieseler

**Affiliations:** 1 Department of Drug Assessment, Institute for Quality and Efficiency in Health Care, Cologne, Germany; 2 Department of Medical Biometry, Institute for Quality and Efficiency in Health Care, Cologne, Germany; National Taiwan University, TAIWAN

## Abstract

The validity of mixed treatment comparisons (MTCs), also called network meta-analysis, relies on whether it is reasonable to accept the underlying assumptions on similarity, homogeneity, and consistency. The aim of this paper is to propose a practicable approach to addressing the underlying assumptions of MTCs. Using data from clinical studies of antidepressants included in a health technology assessment (HTA), we present a stepwise approach to dealing with challenges related to checking the above assumptions and to judging the robustness of the results of an MTC. At each step, studies that were dissimilar or contributed to substantial heterogeneity or inconsistency were excluded from the primary analysis. In a comparison of the MTC estimates from the consistent network with the MTC estimates from the homogeneous network including inconsistencies, few were affected by notable changes; that is, a change in effect size (factor 2), direction of effect or statistical significance. Considering the small proportion of studies excluded from the network due to inconsistency, as well as the number of notable changes, the MTC results were deemed sufficiently robust. In the absence of standard methods, our approach to checking assumptions in MTCs may inform other researchers in need of practical options, particularly in HTA.

## Introduction

Health technology assessment (HTA) agencies such as the German Institute for Quality and Efficiency in Health Care (IQWiG) or the UK National Institute for Health and Care Excellence (NICE) consider mixed treatment comparisons (MTCs) as a method for evidence synthesis in their assessments [1, 2]. The validity of an MTC relies on whether it is reasonable to accept the underlying similarity, homogeneity and consistency assumptions [3, 4], namely, that studies are sufficiently similar concerning moderators of the relevant treatment effect, studies are sufficiently homogeneous to be quantitatively combined, and direct and indirect evidence estimate the same effect [4]. For pairwise meta-analysis, it is common practice to explore the validity of similarity and homogeneity assumptions [5]. In the context of simultaneous analyses of multiple comparisons, checking the validity of all 3 assumptions is equally important. This is best done using a systematic approach.

There is a lack of objective methods to assess and improve clinical similarity of trials [4], while methods to assess statistical homogeneity and consistency in the MTC network are rarely and poorly applied [6]. Guidelines with checklists and further detailed guidance are available to support the reporting and reviewing of MTCs [3, 4, 7–12]. Nevertheless, few published MTCs explicitly compare direct and indirect evidence [4] and the question how to interpret differences in effect estimates as a result of dealing with violated assumptions is rarely addressed. Steps required to develop a study pool appropriate for an MTC analysis need to be discussed.

The aim of this paper is to present a practicable stepwise approach to addressing the underlying assumptions of MTCs and to provide a worked example on how to judge the robustness of results of an MTC. Combining data from clinical studies of antidepressants in an MTC model, we calculated effect estimates for a health economic evaluation. Here we describe our approaches to checking the underlying assumptions, to handling insufficient clinical similarity and statistical heterogeneity between studies, and to handling inconsistency in the MTC network. Specifically, this paper illustrates the steps taken with regard to the creation of the study pool, and discusses our findings within the context of HTA.

It should be noted that the aim of the MTC was to produce treatment effects for the health economic evaluation as the “target analysis”, the basis for decision making. Therefore, instead of splitting data pools and calculating treatment effects for subgroups to draw separate clinical conclusions (e.g. on the subgroup included in studies of all ages vs. the subgroup included in studies of elderly patients only), we only considered studies in the MTC that represented the broader clinical question of the health economic evaluation.

## Methods

The following sections describe the methods of our stepwise approach.

Detailed methods relating to the data set for calculating the MTC are presented in S1 Appendix. In summary, the MTC results presented here are taken from a recent health economic evaluation of four antidepressants (mirtazapine, venlafaxine, duloxetine and bupropion) [13]. The health economic evaluation used clinical data from clinical studies included in two previous benefit assessments to calculate treatment effects to feed into the cost effectiveness calculations. The studies included provided data on a population with a primary diagnosis of major depression in an acute treatment setting. For this paper we use the results of the outcome “treatment discontinuation due to adverse events” as an example. As a statistical model we applied an MTC meta-analysis according to the methods suggested by Lu and Ades and combined direct and indirect evidence within a Bayesian framework [14, 15]. Details are given in S2 Appendix.

For this paper we limit the MTC output of our worked example to the treatment comparisons with placebo. All MTC analyses were conducted on the basis of odds ratios (ORs) using intention-to-treat (ITT) analyses of the studies included. The full set of MTCs can be found in S3 Appendix and in the full report on the health economic evaluation [13]. A full list of all studies considered for the health economic evaluation (n = 138) is included as S4 Appendix.

### Step 1: Clinical similarity

The first step addresses our approach to checking the clinical similarity assumption in the data pool.

In the previous benefit assessments, studies had to fulfil various inclusion criteria (see S1 Appendix). To further judge clinical similarity between studies, we assessed known effect modifiers so that study results could be meaningfully combined in meta-analysis. In terms of population characteristics, specific populations, such as patients with depression as comorbidity, were deemed dissimilar to the population with a primary diagnosis of major depression (in accordance with the previous benefit assessments). As a result, these studies were not analysed within the network of primary interest presented in this paper. In terms of study characteristics, studies with adapted designs and longer follow-up periods appropriate for investigating the prevention of relapse or recurrence of depression were excluded for lack of similarity, as they did not investigate the acute phase treatment.

In addition, we assessed the similarity of the definition of the outcome “treatment discontinuation due to adverse events”, the availability of results for this outcome, and the similarity of the analyses applied. Consequently, study publications that did not report this outcome at all were excluded from the study pool.

### Step 2: Statistical homogeneity

In a second step we applied our approach to checking the homogeneity assumption within each pairwise contrast according to our standard methodology [2] by conducting meta-analyses with random effect models [16]. Heterogeneity was analysed using I^2^ and was classified to be substantial if I^2^ > 50% [17]. If substantial heterogeneity was identified, the studies with potential contributing factors such as predefined effect modifiers (e.g. age, that is, studies only investigating elderly patients) and/or a high risk of bias, were excluded. To limit potential selection bias, studies where no contributing factors could be identified were excluded from the main analysis and the impact of exclusion explored in sensitivity analyses on the level of the MTC analysis. Consequences of study exclusions in the clinical study pool were addressed in sensitivity analyses around the results of the health economic evaluation; the presentation of these analyses is beyond the scope of this paper.

As a result of this approach to checking the homogeneity assumption the adjusted indirect comparisons were based solely on pairwise contrasts without substantial heterogeneity.

### Step 3: Consistency in the network of treatments

Step 3 addresses our approach applied to checking the consistency assumption within the MTC. The MTC network connects treatments via common comparators (e.g. placebo). If studies directly comparing treatments are also included, the network forms a closed loop, which enables checking the consistency assumption. For this purpose, we chose the residual deviance approach suggested by Dias et al. [18]. According to their approach, the study (or study arm for multi-arm studies) with the highest contribution to the poor fit of the model is eliminated from the analyses and the MTC recalculated for exploration purposes. For the approach presented here, this process is then repeated until the network no longer shows inconsistency (residual deviance + leverage ≤ 3 for all study arms) [19], as a robust network was required to calculate effect estimates for the subsequent health economic evaluation.

The MTC treatment effects and their credible intervals were deemed sufficiently robust to be incorporated in the subsequent health economic primary and sensitivity analyses, provided the proportion of studies (or study arms in multi-arm studies) excluded for inconsistency reasons did not exceed the threshold of 20%.

To assess the impact of achieving consistency in the network, we compared the resulting expected treatment effects with the corresponding results on the basis of all studies in the network prior to consistency checking. We considered the following changes as notable: any change in direction of effects or statistical significance as well as more than two-fold changes in effect sizes. As minor changes (e.g. OR 0.98 vs. 1.01) could simply be due to chance, we did not solely present overall numbers but described the changes in detail.

### Step 4: Sensitivity analysis to explore robustness

To assess the robustness of our stepwise approach to checking the homogeneity and consistency assumptions and handling violations, we performed a sensitivity analysis. For this sensitivity analysis, we calculated results from a network for which we had omitted the homogeneity check but had performed the consistency check (i.e. comparison of a consistent network without a homogeneity check vs. one with a homogeneity check). We then compared the studies that had been excluded from the study pools. We also explored any notable changes (see above) in effects and confidence intervals (CIs) between those study pools.

## Results

**Fig 1:**
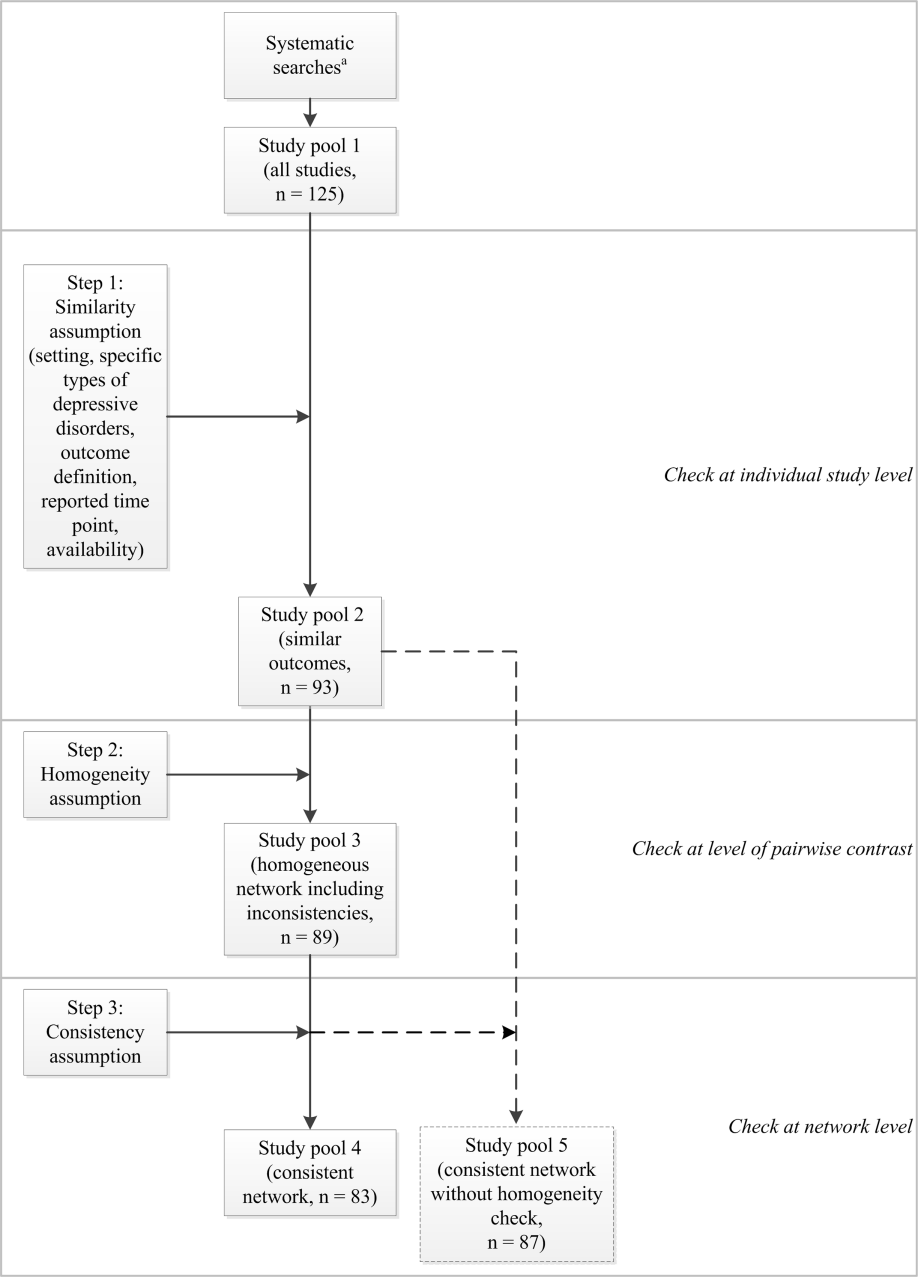
Stepwise approach to create the study pool for MTC analysis.

Fig 1 shows the steps that led to our final study pool for the primary MTC analysis (study pool 4).

Updating the searches for the previous benefit assessments and applying the inclusion and exclusion criteria contributed 125 studies (study pool 1 [all studies]). Fig 1 also shows details of the number of included studies before and after the individual steps for creating the respective study pools.

In the sections below we report the results of our stepwise approach, together with a description of the excluded studies and study arms and resulting study pools. We also explore the resulting effect estimates using the stepwise approach to checking the main assumptions. For the consistency assumption, we present the results of the assessment of notable changes using set criteria. Finally, we present a sensitivity analysis conducted for this publication to check the robustness of our stepwise approach.

### Results of the stepwise approach to checking similarity, homogeneity and consistency

#### Step 1: Achieving similarity and resulting study pool

18 out of the 125 studies (14%) were excluded from study pool 1 (all studies) as they had been carried out in different settings (n = 11) or included mostly or exclusively patients who were treatment resistant (n = 3), were diagnosed following myocardial infarction (n = 1) or had seasonal affective disorder (n = 3). However, results data on treatment discontinuation due to adverse events were not available from all of the remaining studies. Exclusion of a further 14 studies with missing data (13%) left 93 studies in the data set (study pool 2 [similar studies and outcomes], see Fig 1). We ensured similarity of reported outcomes by applying outcome-specific criteria, so that only studies reporting information that was sufficiently similar concerning the outcome definition and analyses reported were included. These outcome-specific similarity criteria were met by all 93 studies.

#### Step 2: Achieving homogeneity and resulting study pool

**Fig 2:**
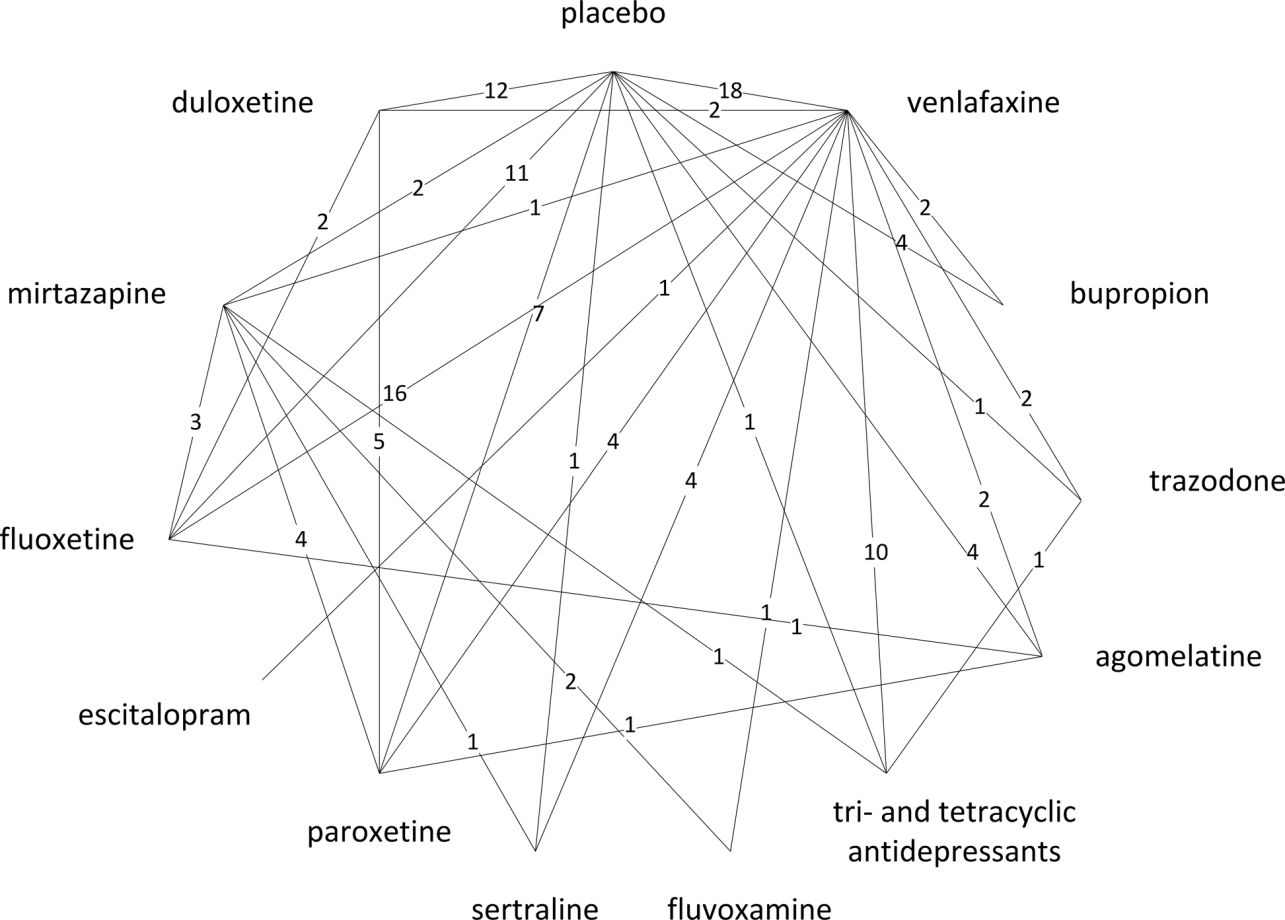
Outcome-specific network (treatment discontinuation due to adverse events, acute studies).

Checking the homogeneity assumption revealed heterogeneity in 4 contrasts. As defined a priori, one drug class (SSRIs) had to be split into 5 individual drugs due to substantial heterogeneity in the mirtazapine-SSRI contrast (I^2^ = 56.2%). In total, 4 out of 93 studies (4%) plus 4 study arms of multi-arm studies were excluded from the study pool due to heterogeneity, namely, 3 studies and 3 arms of multi-arm studies across 2 contrasts with a high risk of bias (venlafaxine-placebo and venlafaxine-escitalopram), as well as 1 study and 1 study arm for the duloxetine-escitalopram contrast for which heterogeneity was unexplained and was addressed by means of a sensitivity analysis. The exclusion of the 4 study arms meant that data on further contrasts were excluded: paroxetine versus placebo, fluoxetine versus placebo, TCAs versus placebo, and escitalopram versus placebo. In total, 89 studies were included in study pool 3 (homogeneous network including inconsistencies, see Fig 1). Fig 2 shows the resulting MTC network of comparators included in the MTC (127 treatment comparisons based on 70 two-arm and 19 three-arm studies, which provided direct evidence on 31 out of 78 possible pairwise contrasts for 1 drug class [TCAs], 11 individual drugs, and placebo).

#### Step 3: Achieving consistency and resulting study pool

Checking the consistency assumption using the deviance information criterion resulted in 6 out of 89 studies (7%) and 2 study arms of three-arm studies being excluded from the data pool of the network in a stepwise fashion. Further details are presented in the full report [13]. As a result, study pool 4 (consistent network) contained 83 studies and formed the data set for the main (primary) results (see Fig 1).

### Exploring effect estimates using the stepwise approach to checking main assumptions

Our approach to judging similarity between studies was based on predefined clinical criteria and concurs with the standard methodology used in systematic reviews [20]. We therefore focus below on exploring the impact of the stepwise approach to dealing with insufficient homogeneity and consistency.

To illustrate variability in MTC estimates, Table 1 shows the treatment effects of comparisons of treatments versus placebo derived from study pool 2 (similar studies and outcomes), study pool 3 (homogeneous network including inconsistencies), study pool 4 (consistent network), and study pool 5 (sensitivity value, consistent network without homogeneity check) of our approach. Please note that results are based on the entire network, but here we limit the illustration to the placebo contrasts. We have included the full set of results in S3 Appendix.

**Table 1:** Change in effect estimates and uncertainty intervals after a stepwise approach to checking homogeneity and consistency assumptions.

Treatment comparison with placebo^a^	Direct pairwise comparisons^b^		Indirect comparison/MTC based on study pool 2 (similar studies and outcomes [network including heterogeneity and inconsistencies])		Indirect comparison/MTC based on study pool 3 (homogeneous network including inconsistencies)		Indirect comparison/MTC based on study pool 4 (consistent network)		Indirect comparison/MTC (sensitivity value^c^, based on study pool 5)	
	n	OR [95% CI]	n	OR [95% CrI]	n	OR [95% CrI]	n	OR [95% CrI]	n	OR [95% CrI]
Agomelatine vs. placebo	4	0.95 [0.47; 1.91]	4	0.89 [0.47; 1.49]	4	0.89 [0.50; 1.47]	4	0.94 [0.53; 1.48]	4	0.90 [0.52; 1.45]
Bupropion vs. placebo	4	1.00 [0.61; 1.65]	4	1.32 [0.75; 2.15]	4	1.33 [0.79; 2.05]	3	1.25 [0.75; 1.95]	3	1.40 [0.86; 2.14]
Duloxetine vs. placebo	12	2.22 [1.55; 3.19]	12	2.87 [2.11; 3.81]	12	2.89 [2.16; 3.80]	10	3.53 [2.66; 4.59]	11	3.24 [2.46; 4.15]
Escitalopram vs. placebo	0	n/a	1	1.02 [0.55; 1.72]	0	1.81 [0.60; 4.22]	0	1.84 [0.71; 3.87]	1	1.17 [0.69; 1.83]
Fluoxetine vs. placebo	11	1.27 [0.88; 1.84]	12	1.41 [1.07; 1.84]	11	1.41 [1.08; 1.82]	10	1.37 [1.07; 1.73]	10	1.33 [1.04; 1.68]
Fluvoxamine vs. placebo	0	n/a	0	1.59 [0.63; 3.31]	0	1.62 [0.68; 3.22]	0	1.55 [0.73; 2.83]	0	1.50 [0.70; 2.79]
Mirtazapine vs. placebo	2	2.75 [1.28; 5.93]	2	2.21 [1.47; 3.22]	2	2.23 [1.53; 3.16]	2	2.18 [1.56; 2.96]	2	2.11 [1.47; 2.89]
Paroxetine vs. placebo	7	2.13 [1.43; 3.17]	8	2.33 [1.68; 3.15]	7	2.40 [1.76; 3.17]	7	2.76 [2.08; 3.59]	8	2.65 [2.00; 3.43]
Sertaline vs. placebo	1	3.36 [1.17; 9.70]	1	1.32 [0.72; 2.19]	1	1.40 [0.81; 2.23]	0	0.77 [0.35; 1.38]	0	0.74 [0.33; 1.41]
Trazodone vs. placebo	1	2.27 [0.95; 5.44]	1	2.59 [1.10; 5.21]	1	2.60 [1.19; 4.96]	1	2.63 [1.27; 4.78]	1	2.55 [1.24; 4.67]
TCAs vs. placebo	1	2.25 [0.88; 5.75]	2	2.45 [1.57; 3.67]	1	2.50 [1.62; 3.68]	1	2.35 [1.56; 3.43]	2	2.28 [1.55; 3.29]
Venlafaxine vs. placebo	18	2.47 [1.81; 3.37]	23	2.20 [1.79; 2.68]	18	2.28 [1.87; 2.79]	17	2.41 [1.99; 2.87]	19	2.33 [1.93; 2.79]

a: To illustrate variability in MTC estimates for study pools 2–5, we restricted this table to show placebo contrasts only; for MTC results in all possible 78 contrasts based on study pools 3 and 4, please see S3 Appendix.

b: Direct estimates based on study pool 3, i.e. after checking the homogeneity assumption.

c: Sensitivity value represents the consistent network before excluding studies due to heterogeneity.

CI: confidence interval, CrI: credible interval, MTC: mixed treatment comparison, n: number of studies contributing to estimate (“0” in the context of MTC-based estimates denotes that the estimate relies solely on indirect evidence), n/a: not applicable (as no relevant studies had been identified for this contrast), OR: odds ratio, TCAs: tri- and tetracyclic antidepressants.

#### Exploring effect estimates after achieving homogeneity

Comparing effects of study pool 2 (similar studies and outcomes) with study pool 3 (homogeneous network including inconsistencies) allows us to explore the degree of change resulting from our method of dealing with heterogeneity.

As a result of heterogeneity, the class of SSRIs had to be split into individual drugs (see above). In return, new pairwise meta-analyses for which heterogeneity was assessed had to be calculated.

The duloxetine-escitalopram contrast showed unexplained heterogeneity (I^2^ = 75.8%), which we addressed by excluding both affected studies in the main analysis and by conducting a sensitivity analysis containing those studies. The effect estimate of the main analysis showed a wide credible interval (CrI) containing the null effect (OR 0.53 [95% CrI 0.19; 1.16]). In the sensitivity analysis, the effect estimate was similar, albeit statistically significant (OR 0.46 [95% CrI 0.26; 0.72]), and both credible intervals overlapped.

The effect estimate of the escitalopram-placebo contrast increased and the credible intervals widened noticeably after removing studies with escitalopram due to heterogeneity. Before the heterogeneity check, the estimate was close to the null effect and not statistically significant. After removing the studies with escitalopram, no direct evidence for the escitalopram-placebo contrast was available and thus this estimate was derived indirectly from the MTC.

Apart from the escitalopram-placebo contrast, all other effect estimates in the placebo contrasts remained more or less equal (no differences in OR ≥ 0.1) and credible intervals narrowed somewhat, reflecting the higher precision after reducing the variability due to heterogeneity.

#### Exploring the effect estimates after achieving consistency

For the whole network, the proportion of studies or study arms excluded to achieve consistency was deemed acceptable. Using data sets from study pools 3 (homogeneous network including inconsistencies) and 4 (consistent network), we qualitatively explored and formally analysed the degree of change in expected treatment effects before and after achieving consistency by excluding studies that contributed to inconsistency (see methods section). This was done by comparing the treatment effects estimated from the consistent network (study pool 4) with those estimated from study pool 3 (homogeneous network including inconsistencies) and describing changes qualitatively, as well as documenting changes in effect size, direction of effect, and statistical significance according to the criteria described in the methods section. As part of our approach we also compared estimates from the consistent network (study pool 4) with the direct effect estimates, where available, using the same criteria to explore notable changes.

Comparing effect estimates of study pool 3 (homogeneous network including inconsistencies) and study pool 4 (consistent network) reveals the impact of excluding studies due to inconsistency. Table 1 shows that after these exclusions, the majority of the 12 placebo contrasts showed more or less similar effect estimates.

#### Notable changes in effect estimates (homogeneous network including inconsistencies vs. consistent network)

Next we report the results of checking the defined criteria for notable changes between estimates of study pools 3 (homogeneous network including inconsistencies) and 4 (consistent network) for all comparisons in the network (see methods section). Table 2 shows the resulting notable changes in direction of effect, effect size, and statistical significance when comparing the effect estimates for each pair of treatments.

**Table 2:** Impact of achieving consistency on expected treatment effects within pairs of treatments in the full network.

Factor considered to assess impact of achieving consistency	Number of notable changes(% of all contrasts)
A. MTC based on study pool 4 (consistent network) vs. MTC based on study pool 3 (homogeneous network including inconsistencies, N = 78)^a^	
Change in direction of effect	7 (9)
Effect size differs by factor 2	3 (4)
Change in statistical significance	7 (9)
B. MTC based on study pool 4 (consistent network) vs. direct effect estimates (all contrasts with direct evidence, N = 31)^a^	
Change in direction of effect	6 (19)
Effect size differs by factor 2	4 (13)
Change in statistical significance	9 (29)

a: The notable changes shown are based on the full MTC network, that is, 89 studies including direct evidence on 31 out of 78 possible pairwise contrasts for 12 drugs plus placebo. For details on the effect and uncertainty estimates, please see S3 Appendix.

Less than 10% of the 78 possible pairwise contrasts were affected by 1 of the 3 notable changes. The full set of estimates for all 78 contrasts in the network is provided in S3 Appendix. There were 7 notable changes in direction of effect. For example, for the sertraline-placebo contrast, the only direct study was excluded from the network and the direction of effect changed (before: sertraline better; after: placebo better). Although the estimates from study pools 3 and 4 were not statistically significant, the direct estimate was, and favoured sertraline (see Table 1). In 6 of the 7 contrasts, the credible intervals of the effect estimates based on study pool 4 (consistent network) contained the effect estimates based on study pool 3 (homogeneous network including inconsistencies). There were 3 more than two-fold changes in effect size. Only in 1 of these did the credible interval contain the effect estimates based on study pool 3 (homogeneous network including inconsistencies). 7 differences in statistical significance occurred between the 2 effect estimates. In all contrasts, the effect estimate was statistically significant after the consistency check (study pool 4 [consistent network]), but not before. In 3 of these the relevant value was close to the null effect.

#### Notable changes in effect estimates (consistent network vs. direct estimates)

Comparing the MTC estimates from the consistent network with the effect estimates from pairwise contrasts based on direct evidence (available for 31 comparisons in the full network, see S3 Appendix), less than a fifth of the MTC estimates from the consistent network showed a change in direction of effect (6 changes, 19%) or a notable change in effect size (4 changes, 13%). There were 9 changes in statistical significance (29%). Of these, 7 were changes from not statistically significant direct estimates to statistically significant MTC estimates, perhaps denoting an increase in precision. The remaining 2 changes occurred in the paroxetine-mirtazapine and the sertraline-placebo contrasts. For the paroxetine-mirtazapine contrast, the direct estimate was based on a meta-analysis of 4 studies, with CIs close to the null effect [OR 1.56 [95% CI 1.01; 2.38]). The credible interval of the MTC estimate was narrower but contained the null effect (1.29 [0.93; 1.75]), so this may be a chance finding. For the sertraline-placebo contrast, the direct estimate was based on a single trial, which was excluded due to inconsistency in the MTC. Not only was the MTC estimate no longer statistically significant, the direction of effect also changed (before: sertraline better; after: placebo better), denoting a disagreement between the direct and indirect evidence in the network. Overall, 6 of the 9 contrasts with changes in statistical significance were “data-poor”, i.e. were based on ≤ 2 direct trials.

In both sets of estimates based on the 2 study pools, the contrasts affected were spread across all comparisons in the network. In 3 of the 6 notable changes in direction of effect the direct ORs were close to the null effect (see S3 Appendix).

We used the same approach to assess the robustness of the MTC results from the sensitivity analyses performed for the health economic evaluation (unexplained heterogeneity, alternative prior distributions), which are not shown in this paper but presented in the full report [13]. In summary, the criteria checked (resulting changes in effect size, direction of effect, and statistical significance) did not indicate sensitivity to the inclusion of both escitalopram-studies (see above) or the choice of an uninformative prior for the primary analysis.

As shown above, more studies were excluded for dissimilarity reasons than as a result of heterogeneity and inconsistency combined. Considering the proportion of studies excluded for inconsistency reasons, as well as the number of notable changes, the data based on study pool 4 (consistent network) was deemed acceptable for use in further analyses.

### Sensitivity analysis: impact of the stepwise approach

As a sensitivity analysis for this paper we calculated results based on study pool 5 (consistent network without a homogeneity check), which we compared with study pool 4 (consistent network), so that the impact of omitting step 2 (homogeneity check) but executing step 3 (consistency check) on effect estimates could be explored. As a result, 6 of the 93 studies in study pool 2 (6%) were excluded and the resulting study pool 5 comprised 87 studies. Following the consistency check, 6 studies and 4 study arms were excluded from study pool 5 (see Fig 1). In contrast, 4 studies and 4 study arms in the main analysis were excluded due to heterogeneity and 6 studies and 2 study arms due to inconsistency (1 study overlapping with 1 study arm was excluded due to heterogeneity), resulting in a total of 10 excluded studies and 5 study arms (see Table 3).

**Table 3:** Agreement of excluded studies and study arms using the proposed stepwise approach and omitting the homogeneity check.

	Primary analysis (step 2 and step 3)		Sensitivity analysis
Study	Study or study arm^a^ excluded due to heterogeneity (step 2 yielding study pool 3)	Study or study arm^a^ excluded due to inconsistency (step 3 yielding study pool 4)	Studies excluded due to inconsistency, omitting step 2 (yielding study pool 5)
0600C1-217-US-CSR-45150	●		●
0600B1-384-US-EU-CA-CSR-41642	●		●
0600B-367-EU-GMR-25782	● (placebo arm)		
0600A1-372-US-GMR-32822	● (placebo arm)		● (placebo arm)
Lecrubier 1997	● (placebo arm)		
Khan 2007	●		
Nierenberg 2007	● (escitalopram arm)	●	
Bielski 2004	●		
Rudolph 1999		● (fluoxetine arm)	● (fluoxetine arm)
Clerc 1994		●	●
Raskin 2007		●	●
0600A-321-GMR-18105		●	●
0600C1-402-US-CA-CSR-48579		● (sertraline arm)	● (sertraline arm)
0600A1-349-NE-UK-CSR		●	●
WXL101497		●	● (placebo arm)
Total excluded evidence	10 studies, 5 study arms^a^		6 studies, 4 study arms^a^
Total evidence in the network	83 studies		87 studies

a: In multi-arm studies, exclusion of single study arms in each step does not lead to exclusion of the study as one or more other direct contrasts remain in the study pool.

● Denotes studies that were affected by described step or analysis.

Table 3 shows that all studies affected were the same studies (or individual study arms of these) as those excluded from the primary analysis following steps 2 and 3. On the other hand, not all studies excluded in the full stepwise approach were excluded in the sensitivity analysis.

The resulting effect estimates based on study pools 4 and 5 were similar in all contrasts (i.e. no notable changes; see Table 1, last 2 columns). In study pools 2 and 3, i.e. before and after homogeneity checking, but both before consistency checking, all contrasts showed either agreement in the direction in which results differed, or similar effect estimates.

## Discussion

### Summary of main findings

We conducted an MTC with the goal to provide estimates for a health economic evaluation and present a worked example here for a stepwise approach to checking the assumptions underlying MTC. We also describe a possible method for dealing with dissimilarity, heterogeneity and inconsistency in the data. We had to deal with issues arising in this specific HTA context and, to the best of our knowledge, our approach has not been applied before. Our experience with the approach showed that, using preselected criteria, it can be applied in a systematic and transparent way and provides a means to explore the robustness of the MTC estimates. It is not restricted to large data sets, but can also be used for smaller study networks.

### Previous research

Investigation of the similarity, homogeneity and consistency assumptions has been described as essential when conducting MTC [3, 4, 7, 21]. Various approaches have been suggested to address these assumptions. To address the similarity assumption, probable moderators of relative treatment effects (clinical, methodological) should be identified. To assess study similarity in adjusted indirect comparisons, study-specific inclusion and exclusion criteria, baseline patient characteristics and study characteristics can be compared within and between studies [8]. Differences in such moderators can lead to heterogeneity; in this case, even a random effects model, which does not assume a fixed treatment effect across studies for a particular pairwise contrast, would be inappropriate. Methods for investigating heterogeneity in standard meta-analysis can be adopted, including subgroup analysis and meta-regression [4]. However, objective and validated methods to assess or improve study similarity are lacking [4], and “there is no commonly accepted standard defining which studies are `similar´ enough” [22].

Established methods to measure heterogeneity in pairwise meta-analysis exist: various researchers have described how to address heterogeneity in the context of MTC using meta-regression and bias adjustment (e.g. [20, 23]). To address the consistency assumption, tests based on the Bucher method for single loops of evidence may be applied [24], which has been extended to multiple loops and methods suitable for more complex networks [25]. However, the bias-adjustment method to address existing heterogeneity is considered ‘semi-experimental’ and further research on internal bias is needed [23]. Moreover, there is little work on how to practically respond to heterogeneity or inconsistency in MTC once it has been identified [19, 26]. One particular problem is addressed by our stepwise approach, namely, the greater the degree of between-study heterogeneity, the lower the detectability of inconsistency [25].

Several questions on the practical use of available methods remain, for example in the context of HTA. What if inconsistency has been identified, but its cause has not? When should this violation of the consistency assumption lead to abandonment of the MTC altogether? To what extent should causes of heterogeneity in pairwise data be explored (e.g. by subgroup analyses or meta-regression) before it can be incorporated in MTC? Both Donegan et al. and Li et al. have described these unanswered questions in detail [6, 27].

Due to the lack of standard approaches for dealing with dissimilarity, heterogeneity, and inconsistency in MTC, we were unable to compare our results with other research findings; thus some uncertainties remain. However, some components of our approach have been described and used elsewhere, such as the method to identify inconsistency [18]. The criteria used for exploring the impact of changes to the data set have in part also been used elsewhere, for example, RoR > 2 featured as a threshold to classify high inconsistency in the work by Chaimani et al. [28].

Other researchers have proposed additional concepts and features of MTC models, such as network geometry and asymmetry [29] as well as alternative methods for individual steps of our proposed approach. To address heterogeneity, additional suggestions include modelling of heterogeneity variance [30] as well as meta-regression (e.g. integrated subgroup analysis) and bias (covariate) adjustment methods [23].

To address inconsistency, additional suggestions include alternative modelling of variance [30], modelling other effect measures and assessing their impact on inconsistency [31], performing sensitivity analyses after covariate adjustments to deal with inconsistency [26], and performing meta-regression [32].

To address multiple assumptions, methods proposed in the literature include using study-level covariates to improve similarity and consistency [33, 34] as well as adding treatment-by-covariate interactions to reduce inconsistencies and explore heterogeneity [35].

Currently there is not one superior alternative for addressing the underlying assumptions of MTC, and all have their own limitations.

### Handling methodological challenges

We excluded studies from the network that contributed to heterogeneity in pairwise contrasts; this is required, as pooling heterogeneous results is not reasonable [20, 36] and may even mask inconsistency in the context of MTC [19], perhaps due to the fact that similar mechanisms may underpin both heterogeneity and inconsistency [23, 31]. However, the exclusion of studies has its disadvantages (e.g. post-hoc adjustments may lead to bias or information may be lost from three-arm studies where one contrast violates the assumption of homogeneity or consistency). We therefore set a threshold for the proportion of studies (20%) below which we considered the validity of the results of the MTC not to be at risk, but one could criticize this threshold as arbitrary. Instead of calculating thresholds for the proportion of studies from the whole network, proportions could be calculated per comparison. As another alternative, a threshold for the maximum proportion of excluded patients could be set. It is also possible to apply study weights based on standard errors, a method used in pairwise meta-analyses [37]. This option would require the calculation of study weights within the adjusted indirect comparisons. In this context it might also be of interest to explore how the network evolves graphically, particularly when presented with networks supported by relatively few studies.

To account for study exclusions, we also explored the impact of changes to the data set on effect estimates in order to judge whether or not the results could be deemed sufficiently robust for further analysis. For this purpose, we explored actual changes in effect estimates between pairwise meta-analyses and MTC results, which may differ unpredictably and thereby influence conclusions on the relative effectiveness of treatments [38]. It seemed important to explore these changes and increase our confidence in the appropriateness of the network. We found that estimates based on “data-poor” contrasts, that is, pairwise contrasts for which little or no direct evidence was available (either because no head-to-head studies existed or due to study exclusions) tended to be more prone to change than “data-rich” contrasts. Other researchers have also found that effect estimates based on “data-poor” contrasts tend to be more heavily influenced by the indirect evidence in the network [28]. Repetition of our approach in a data-poor situation is recommended as further analysis; it is expected that results would vary a lot more than in a data-rich situation if studies were excluded. In our context we found our stepwise approach was suitable to provide a systematic guide and transparent basis for sensitivity analysis or to enable a judgement as to whether or not results represented a sufficiently robust basis for decision making. However, the potential disadvantages mentioned above need to be critically discussed.

We chose to exclude individual studies and/or study arms if they were found to contribute to substantial heterogeneity or inconsistency; alternatively, it would have been possible to exclude the entire network node, i.e. the comparator treatment. However, this approach has been shown to potentially introduce relevant bias [39]. It would also be equally possible to use the inconsistency check to identify and exclude contributing studies for sensitivity analysis only, so as not to lose evidence from the main network. However, due to the stepwise approach presented here this would be made explicit and inform the sensitivity analysis that is required to address the consistency assumption.

We also explored the impact of using a stepwise approach to dealing with heterogeneity and inconsistency and found that the studies excluded in either step of the primary analysis largely overlapped with those excluded in the sensitivity analysis. This practical example supports the idea that similar mechanisms underpin the concepts of heterogeneity and inconsistency (see above). However, inconsistency and heterogeneity may not always coexist [6]. In addition, adapting the data set in this way constitutes a post-hoc type of analysis. Whilst not all effect modifiers on a study or network level are known, any violations of assumptions have to be dealt with. Even so, our approach yields a homogeneous study pool for synthesis, which has been described as a prerequisite for estimates of pairwise data for which no head-to-head studies yet exist [36].

Uncertainty in effect estimates as a basis for decision making should be thoroughly explored. Instead of providing a basis for drawing conclusions on clinical effectiveness, the aim of this MTC was to generate treatment effect estimates in order to inform a subsequent health economic evaluation. The uncertainty in treatment effect estimates, including the impact of study exclusions on the results and conclusions of the cost-effectiveness analyses, was explored and presented in the health economics report [13]. We focus here on presenting means of checking the robustness of results for subsequent analysis, instead of discussing the impact on cost-effectiveness conclusions, as this was not the aim of the present work.

### Limitations

The MTC study pool we used for this analysis contained 89 studies and was thus comparatively large; HTAs in other therapeutic indications may contain fewer studies. In MTCs of such HTAs, the impact of our stepwise approach on the estimated treatment effects might be greater. Although the stepwise approach is also feasible in situations where study pools are smaller [13], we did not examine the transferability of our findings to other HTA settings. Furthermore, as noted above, we were unable to compare our approach to a reference approach. Further explorations using simulations, for example, were beyond the scope of this analysis. We defined a priori which test statistics for heterogeneity and indices for inconsistency we would use. While we would expect established statistics to detect heterogeneity if present, it is unclear how the use of alternative indices, particularly relating to inconsistency, would affect results.

### Conclusion

We present a systematic and transparent approach to dealing with the 3 central assumptions for MTC. Even though choices had to be made to judge the robustness of the data after adapting the evidence base, the approach applied provides transparency at each step of the analysis and exploration of differences by means of sensitivity analysis. In the absence of standard methods, our approach may inform other researchers in need of practical options, particularly in HTA. However, the validity of effect estimates resulting from our approach has to be further evaluated and further research on the comparison of practical approaches is needed.

## Supporting Information

S1 Appendix Data set. (DOCX)

S2 Appendix Statistical methods for mixed treatment comparisons. (DOCX)

S3 Appendix Full results. (DOCX)

S4 Appendix Full list of studies. (DOCX)
