# Supplementary material for: Assumptions of Mixed Treatment Comparisons in Health Technology Assessments - Challenges and Possible Steps for Practical Application
Source: PLoS One. 2016 Aug 10;11(8):e0160712. doi: 10.1371/journal.pone.0160712 (PMC4979893; doi:10.1371/journal.pone.0160712)
Supplement: S1 Appendix — (DOCX) [file pone.0160712.s001.docx]

**S1 Appendix: data set**

The data set underpinning the presented analyses was based on clinical studies included in 2 benefit assessments of antidepressants [1,2]. The benefit assessments included short-term (at least 6 weeks) and long-term (at least 6 months) randomized controlled trials (RCTs) of the newer antidepressants venlafaxine, duloxetine, bupropion, mirtazapine, and reboxetine versus placebo or other drugs for major depression in adults. Data from the clinical studies identified were used to inform a health economic evaluation [3]. As the health economic evaluation sought to compare as many relevant treatments for depression as possible the aim of the clinical data synthesis was to obtain treatment effects from the entire treatment network. Consequently, the treatment effects from the MTC were not used to make judgements on clinical effectiveness per se, but to feed into an economic evaluation as the target analysis. Uncertainty surrounding treatment effects derived from the MTC were therefore explored on an MTC level as well as on the level of the target analysis, the health economic evaluation. For this purpose, sensitivity analyses were conducted on all levels of analysis.

For the health economic evaluation, the systematic literature searches of the benefit assessments were updated using appended exclusion criteria to reflect the then current reimbursement and prescribing status (e.g. reboxetine had been excluded from reimbursement in Germany due to a lack of benefit [4] and was thus not considered in the present analysis). The full search strategy and inclusion criteria for the studies included in the MTC are described in full in the above-mentioned benefit assessments and health economic evaluation, which are available online. Searches for studies of the 4 antidepressants under review (target drugs) yielded evidence on comparisons with selective serotonin reuptake inhibitors (SSRIs: citalopram, escitalopram, fluoxetine, fluvoxamine, paroxetine, and sertraline), as well as tricyclic and tetracyclic antidepressants (TCAs: amitriptyline, clomipramine, dosulepin, imipramine, nortriptyline, and maprotiline), agomelatine, and trazodone. According to the protocol of the health economic evaluation, TCAs and SSRIs were initially included as a drug class, but were to be split into individual drugs if substantial heterogeneity was identified [4]. Additional searches for data on comparisons that did not contain 1 of the 4 target drugs were not performed.

In the benefit assessments, outcome-specific networks were analysed separately by MTC. For our current analysis, from these MTCs we selected the binary outcome “treatment discontinuation due to adverse events” from short-term studies with a minimum duration of 6 weeks investigating the treatment of acute depression, as this MTC included a relatively large network posing a wide range of methodological challenges.

These challenges related to insufficient similarity of study characteristics within the available study pool, heterogeneity in pairwise comparisons (contrasts), and inconsistency across all studies and comparisons in the network. For this paper we limit the output of our worked example (Table 1) to the treatment comparisons with placebo. The full set of comparisons can be found in S3 Appendix and in the full report on the health economic evaluation [3]. A full list of all studies considered for the health economic evaluation (n=138) is included as SIV Appendix.

All analyses were conducted on the basis of odds ratios (ORs) using intention-to-treat (ITT) analyses of the studies included.

**References**

1. Institut für Qualität und Wirtschaftlichkeit im Gesundheitswesen (2009a) Selektive Serotonin- und Noradrenalin-Wiederaufnahmehemmer (SNRI) bei Patienten mit Depressionen: Abschlussbericht; Auftrag A05-20A. Available: <http://www.iqwig.de/download/A05-20A_Abschlussbericht_SNRI_bei_Patienten_mit_Depressionen.pdf>. Accessed 11.03.2013.

2. Institut für Qualität und Wirtschaftlichkeit im Gesundheitswesen (2009b) Bupropion, Mirtazapin und Reboxetin bei der Behandlung der Depression: Abschlussbericht; Auftrag A05-20C. Available: <https://www.iqwig.de/download/A05-20C_Abschlussbericht_Bupropion_Mirtazapin_und_Reboxetin_bei_Depressionen.pdf>. Accessed 11.03.2013.

3. Institut für Qualität und Wirtschaftlichkeit im Gesundheitswesen (2013) Kosten-Nutzen-Bewertung von Venlafaxin, Duloxetin, Bupropion und Mirtazapin im Vergleich zu weiteren verordnungsfähigen medikamentösen Behandlungen: Abschlussbericht; Auftrag G09-01. Available: <https://www.iqwig.de/download/G09-01_Abschlussbericht_Kosten-Nutzen-Bewertung-von-Venlafaxin-Duloxetin....pdf>. Accessed 30.10.2013.

4. Institut für Qualität und Wirtschaftlichkeit im Gesundheitswesen (2011) Kosten-Nutzen-Bewertung von Venlafaxin, Duloxetin, Bupropion und Mirtazapin im Vergleich zu weiteren verordnungsfähigen medikamentösen Behandlungen: Berichtsplan (vorläufige Version); Auftrag G09-01. Available: <http://www.iqwig.de/download/G09-01_vorlaeufiger_Berichtsplan_KNB_von_Venlafaxin_Duloxetin_Bupropion_und_Mirtazapin.pdf>. Accessed 11.03.2013.
