# Supplementary material for: Assumptions of Mixed Treatment Comparisons in Health Technology Assessments - Challenges and Possible Steps for Practical Application
Source: PLoS One. 2016 Aug 10;11(8):e0160712. doi: 10.1371/journal.pone.0160712 (PMC4979893; doi:10.1371/journal.pone.0160712)
Supplement: S2 Appendix — (DOCX) [file pone.0160712.s002.docx]

**S2 Appendix: statistical methods for mixed treatment comparisons**

For a simultaneous analysis of all relevant treatments, we used MTC meta-analysis according to the methods suggested by Lu and Ades and combined direct and indirect evidence within a Bayesian framework [1,2]. Suppose placebo is chosen as the overall MTC reference intervention for relative effects denoted as $A$. This means that the effect parameters $d_{Ak}$of all other interventions $k$ versus placebo are modelled directly as basic parameters. We use uninformative priors assumed to be normally distributed, i.e.

$$d_{Ak}\sim N\left( 0,1000 \right) \text{for} k\in T, T=\left\{ all other relevant interventions \right\}.$$

Assuming consistency within the network, the effect parameters of all active interventions out of set $T$ can then each be calculated as functional parameters by

$$d_{xy}=d_{Ay}-d_{Ax} \text{for} x,y\in T, x\neq y.$$

If there is no single treatment *A* to which all other treatments have been compared, any subset of effect parameters can also be chosen as basic parameters, as long as the functional parameters can be written as a linear relation of the basic parameters.

For study $j$, outcome counts for a binary outcome for intervention $k$ are summarized by the number of events $r_{jk}$ out of the number of patients at risk $n_{jk}.$The number $r_{jk}$ is assumed to follow a binomial distribution with parameters $p_{jk}$ and $n_{jk}$, whereas $p_{jk}$ is modeled by a logistic regression model. For each study $j$, a study-specific baseline log-odds $\mu_{jb}$ of intervention $b$ is assumed, together with the log-odds ratio $\delta_{jbk}$ of the outcome for intervention $k$ relative to $b$, i.e.,

$$r_{jk}\sim Bin\left( p_{jk}, n_{jk} \right)$$

$\text{logit}\left( p_{jk} \right)= \left\{ \begin{matrix} \mu_{jb} \\ \mu_{jb}+\delta_{jbk} \end{matrix}\begin{matrix} b\in\text{T}\text{ for} k=b \\ \text{for} k \text{>} b \end{matrix} \right.$.

Study-specific $\delta_{jbk}\sim N({(d}_{Ak}-d_{Ab}), \sigma^{2})$ are derived from a random effects model with a mean log-odds ratio $d_{bk}=d_{Ak}-d_{Ab}$ and a homogeneous variance $\sigma^{2}$. For multi-arm studies we consider a multivariate normal distribution of $\delta_{jbk}$ with a covariance of $\sigma^{2}/2,$ reflecting the assumption of a homogeneous variance in all arms [2]. For study baseline values and basic parameters, vague prior distributions $\mu_{jb}\sim N\left( 0, 1000 \right)$ and $d_{Ak}\sim N\left( 0, 1000 \right)$ are specified. Between-study variance $\sigma^{2}$ is assumed to follow an uninformative uniform distribution $\sigma\sim Uniform (0, 2)$. To check the robustness of the results of the main analysis, 2 sensitivity analyses with alternative vague prior distributions were conducted. The results proved to be robust against alternative prior choices. The full report provides further details on the methods and results of these sensitivity analyses [3].

We assume that the treatments considered are coded in numerical order, so that $k>b$ indicates that treatment $k$ is compared to baseline treatment $b$ of that study. The coding of baseline treatment $b$ is arbitrary; it is assigned a lower code than the code(s) of the other treatment arms in a particular study. This ensures *A* to be the reference treatment in all studies if study arms including *A* are present.

We implemented the model into BUGS using the software OpenBUGS [4] and used 3 chains with a burn-in of 100 000 followed by 50 000 updates to obtain posterior estimates. Convergence was assessed by the Brooks-Gelman-Rubin method [5,6] and by visual inspection of the history plots. Calculations were performed in R 2.12.0 [7] using the library BRugs 0.5-3 [8] for the connection to OpenBUGS.

**References**

1. Lu G, Ades AE (2004) Combination of direct and indirect evidence in mixed treatment comparisons. Stat Med 23: 3105-3124.

2. Lu GB, Ades AE (2006) Assessing evidence inconsistency in mixed treatment comparisons. J Am Stat Assoc 101: 447-459.

3. Institut für Qualität und Wirtschaftlichkeit im Gesundheitswesen (2013) Kosten-Nutzen-Bewertung von Venlafaxin, Duloxetin, Bupropion und Mirtazapin im Vergleich zu weiteren verordnungsfähigen medikamentösen Behandlungen: Abschlussbericht; Auftrag G09-01. Available: <https://www.iqwig.de/download/G09-01_Abschlussbericht_Kosten-Nutzen-Bewertung-von-Venlafaxin-Duloxetin....pdf>. Accessed 30.10.2013.

4. Lunn D, Spiegelhalter D, Thomas A, Best N (2009) The BUGS project: evolution, critique and future directions. Stat Med 28: 3049-3067.

5. Brooks S, Gelman A (1998) General methods for monitoring convergence of iterative simulations. Journal of Computational and Graphical Statistics 7: 434-455.

6. Gelman A, Rubin DB (1992) Inference from iterative simulation using multiple sequences. Stat Sci 7: 457-472.

7. R Development Core Team (2009) R: A language and environment for statistical computing. Vienna, Austria: R Foundation for Statisical Computing. Available: <http://www.R-project.org/>.

8. Thomas A, O’Hara B, Ligges U, Sturtz S (2006) Making BUGS open. R News 6: 12-17.
