# Supplementary material for: Assumptions of Mixed Treatment Comparisons in Health Technology Assessments - Challenges and Possible Steps for Practical Application
Source: PLoS One. 2016 Aug 10;11(8):e0160712. doi: 10.1371/journal.pone.0160712 (PMC4979893; doi:10.1371/journal.pone.0160712)
Supplement: S3 Appendix — (DOCX) [file pone.0160712.s003.docx]

**S3 Appendix:** **Table (full results)**

Table: Treatment discontinuation due to adverse events (acute studies), results: direct comparison; MTC (study pool 4 [all studies – homogeneous network including inconsistencies])/study pool 5 [consistent network])

Table. “Treatment discontinuation due to adverse events” (acute studies), results: direct comparison; MTC (study pool 4 [all studies – homogeneous network including inconsistencies])/study pool 5 [consistent network])

|  |  |  |  | MTC^a^ | |
| --- | --- | --- | --- | --- | --- |
|  | Drug comparisons | Studies | Direct comparison  OR [95% CI] | Study pool 4 (All studies – homogeneous network including inconsistencies)  OR [95% CrI] | Study pool 5 (consistent network)  OR [95% CrI] |
|  | DIC |  |  | 197,18 | 167,31 |
| Placebo | Duloxetine vs. placebo | 12 | 2.22 [1.55; 3.19] | 2.89 [2.16; 3.80] | 3.53 [2.66; 4.59] |
|  | Venlafaxine vs. placebo | 18 | 2.47 [1.81; 3.37] | 2.28 [1.87; 2.79] | 2.41 [1.99; 2.87] |
|  | Mirtazapine vs. placebo | 2 | 2.75 [1.28; 5.93] | 2.23 [1.53; 3.16] | 2.18 [1.56; 2.96] |
|  | Bupropion vs. placebo | 4 | 1.00^b^ [0.61; 1.65] | 1.33 [0.79; 2.05] | 1.25 [0.75; 1.95] |
|  | Fluoxetine vs. placebo | 11 | 1.27 [0.88; 1.84] | 1.41 [1.08; 1.82] | 1.37 [1.07; 1.73] |
|  | Escitalopram vs. placebo |  |  | 1.81 [0.60; 4.22] | 1.84 [0.71; 3.87] |
|  | Paroxetine vs. placebo | 7 | 2.13 [1.43; 3.17] | 2.40 [1.76; 3.17] | 2.76 [2.08; 3.59] |
|  | Sertraline vs. placebo | 1 | 3.36 [1.17; 9.70] | 1.40 [0.81; 2.23] | 0.77 [0.35; 1.38] |
|  | Fluvoxamine vs. placebo |  |  | 1.62 [0.68; 3.22] | 1.55 [0.73; 2.83] |
|  | TCAs vs. placebo | 1 | 2.25 [0.88; 5.75] | 2.50 [1.62; 3.68] | 2.35 [1.56; 3.43] |
|  | Agomelatine vs. placebo | 4 | 0.95 [0.47; 1.91] | 0.89 [0.50; 1.47] | 0.94 [0.53; 1.48] |
|  | Trazodone vs. placebo | 1 | 2.27 [0.95; 5.44] | 2.60 [1.19; 4.96] | 2.63 [1.27; 4.78] |
| Duloxetine | Venlafaxine vs. duloxetine | 2 | 0.56 [0.36; 0.86] | 0.80 [0.59; 1.07] | 0.69 [0.52; 0.91] |
|  | Mirtazapine vs. duloxetine |  |  | 0.78 [0.50; 1.17] | 0.63 [0.42; 0.90] |
|  | Bupropion vs. duloxetine |  |  | 0.47 [0.26; 0.76] | 0.36 [0.20; 0.60] |
|  | Fluoxetine vs. duloxetine | 2 | 0.60 [0.19; 1.92] | 0.50 [0.35; 0.69] | 0.39 [0.28; 0.54] |
|  | Escitalopram vs. duloxetine |  |  | 0.64 [0.20; 1.51] | 0.53 [0.19; 1.16] |
|  | Paroxetine vs. duloxetine | 5 | 0.76 [0.50; 1.15] | 0.84 [0.59; 1.15] | 0.79 [0.58; 1.07] |
|  | Sertraline vs. duloxetine |  |  | 0.49 [0.27; 0.82] | 0.22 [0.10; 0.42] |
|  | Fluvoxamine vs. duloxetine |  |  | 0.57 [0.23; 1.14] | 0.45 [0.21; 0.83] |
|  | TCAs vs. duloxetine |  |  | 0.88 [0.53; 1.36] | 0.68 [0.43; 1.04] |
|  | Agomelatine vs. duloxetine |  |  | 0.31 [0.17; 0.54] | 0.27 [0.15; 0.44] |
|  | Trazodone vs. duloxetine |  |  | 0.92 [0.40; 1.82] | 0.76 [0.35; 1.44] |
| Venlafaxine | Mirtazapine vs. venlafaxine | 1 | 0.69 [0.37; 1.28] | 0.98 [0.68; 1.36] | 0.91 [0.66; 1.22] |
|  | Bupropion vs. venlafaxine | 2 | 0.83 [0.43; 1.61] | 0.59 [0.34; 0.91] | 0.52 [0.30; 0.83] |
|  | Fluoxetine vs. venlafaxine | 16 | 0.67 [0.54; 0.83] | 0.62 [0.49; 0.77] | 0.57 [0.46; 0.70] |
|  | Escitalopram vs. venlafaxine | 1 | 0.71 [0.32; 1.56] | 0.79 [0.27; 1.82] | 0.76 [0.30; 1.59] |
|  | Paroxetine vs. venlafaxine | 4 | 0.93 [0.50; 1.72] | 1.06 [0.77; 1.40] | 1.15 [0.83; 1.49] |
|  | Sertraline vs. venlafaxine | 4 | 0.77 [0.47; 1.25] | 0.62 [0.36; 0.96] | 0.32 [0.15; 0.57] |
|  | Fluvoxamine vs. venlafaxine | 1 | 1.34 [0.30; 6.02] | 0.71 [0.30; 1.42] | 0.65 [0.31; 1.18] |
|  | TCAs vs. venlafaxine | 10 | 1.03 [0.71; 1.49] | 1.10 [0.74; 1.56] | 0.98 [0.68; 1.38] |
|  | Agomelatine vs. venlafaxine | 2 | 0.27 [0.13; 0.55] | 0.39 [0.22; 0.64] | 0.39 [0.22; 0.61] |
|  | Trazodone vs. venlafaxine | 2 | 1.11 [0.56; 2.22] | 1.15 [0.53; 2.17] | 1.09 [0.53; 1.97] |

(continued)

Table: “Treatment discontinuation due to adverse events” (acute studies), results: direct comparison; MTC (study pool 4 [all studies – homogeneous network including inconsistencies])/study pool 5 [consistent network]) (continued)

|  |  |  |  | MTC^a^ | |
| --- | --- | --- | --- | --- | --- |
|  | Drug comparisons | Studies | Direct comparison  OR [95% CI] | Study pool 4 (All studies– homogeneous network including inconsistencies)  OR [95% CrI] | Study pool 5 (consistent network)  OR [95% CrI] |
| Mirtazapine | Bupropion vs. mirtazapine |  |  | 0.62 [0.32; 1.04] | 0.59 [0.32; 0.99] |
|  | Fluoxetine vs. mirtazapine | 3 | 0.55 [0.31; 0.97] | 0.65 [0.44; 0.93] | 0.64 [0.45; 0.87] |
|  | Escitalopram vs. mirtazapine |  |  | 0.83 [0.26; 2.02] | 0.86 [0.31; 1.88] |
|  | Paroxetine vs. mirtazapine | 4 | 1.56 [1.01; 2.38] | 1.10 [0.74; 1.55] | 1.29 [0.93; 1.75] |
|  | Sertraline vs. mirtazapine | 1 | 0.23 [0.08; 0.61] | 0.64 [0.35; 1.06] | 0.36 [0.17; 0.64] |
|  | Fluvoxamine vs. mirtazapine | 2 | 0.60 [0.31; 1.18] | 0.73 [0.33; 1.36] | 0.71 [0.36; 1.26] |
|  | TCAs vs. mirtazapine | 1 | 0.95 [0.06; 15.54] | 1.15 [0.67; 1.84] | 1.10 [0.67; 1.72] |
|  | Agomelatine vs. mirtazapine |  |  | 0.41 [0.21; 0.73] | 0.44 [0.24; 0.73] |
|  | Trazodone vs. mirtazapine |  |  | 1.20 [0.51; 2.42] | 1.23 [0.56; 2.37] |
| Bupropion | Fluoxetine vs. bupropion |  |  | 1.13 [0.65; 1.87] | 1.16 [0.67; 1.95] |
|  | Escitalopram vs. bupropion |  |  | 1.44 [0.43; 3.66] | 1.55 [0.53; 3.57] |
|  | Paroxetine vs. bupropion |  |  | 1.92 [1.07; 3.22] | 2.35 [1.31; 3.90] |
|  | Sertraline vs. bupropion |  |  | 1.12 [0.53; 2.09] | 0.65 [0.25; 1.34] |
|  | Fluvoxamine vs. bupropion |  |  | 1.29 [0.47; 2.85] | 1.32 [0.51; 2.74] |
|  | TCAs vs. bupropion |  |  | 1.99 [1.04; 3.54] | 1.99 [1.05; 3.43] |
|  | Agomelatine vs. bupropion |  |  | 0.71 [0.33; 1.37] | 0.80 [0.36; 1.48] |
|  | Trazodone vs. bupropion |  |  | 2.08 [0.81; 4.48] | 2.24 [0.89; 4.65] |
| Fluoxetine | Escitalopram vs. fluoxetine |  |  | 1.29 [0.43; 3.04] | 1.35 [0.52; 2.91] |
|  | Paroxetine vs. fluoxetine |  |  | 1.73 [1.19; 2.38] | 2.04 [1.47; 2.75] |
|  | Sertraline vs. fluoxetine |  |  | 1.00^c^ [0.56; 1.62] | 0.57 [0.26; 1.03] |
|  | Fluvoxamine vs. fluoxetine |  |  | 1.16 [0.48; 2.31] | 1.14 [0.53; 2.10] |
|  | TCAs vs. fluoxetine |  |  | 1.79 [1.13; 2.67] | 1.73 [1.12; 2.58] |
|  | Agomelatine vs. fluoxetine | 1 | 1.03 [0.20; 5.20] | 0.64 [0.35; 1.07] | 0.69 [0.38; 1.10] |
|  | Trazodone vs. fluoxetine |  |  | 1.87 [0.83; 3.63] | 1.94 [0.93; 3.56] |
| Escitalopram | Paroxetine vs. escitalopram |  |  | 1.70 [0.55; 4.03] | 1.81 [0.68; 3.98] |
|  | Sertraline vs. escitalopram |  |  | 0.98 [0.29; 2.46] | 0.51 [0.15; 1.33] |
|  | Fluvoxamine vs. escitalopram |  |  | 1.14 [0.27; 3.16] | 1.02 [0.29; 2.58] |
|  | TCAs vs. escitalopram |  |  | 1.76 [0.55; 4.26] | 1.54 [0.56; 3.46] |
|  | Agomelatine vs. escitalopram |  |  | 0.62 [0.18; 1.60] | 0.62 [0.20; 1.46] |
|  | Trazodone vs. escitalopram |  |  | 1.82 [0.46; 4.94] | 1.71 [0.53; 4.25] |

(continued)

Table: “Treatment discontinuation due to adverse events” (acute studies), results: direct comparison; MTC (study pool 4 [all studies – homogeneous network including inconsistencies])/study pool 5 [consistent network]) (continued)

|  |  |  |  | MTC^a^ | |
| --- | --- | --- | --- | --- | --- |
|  | Drug comparisons | Studies | Direct comparison  OR [95% CI] | Study pool 4 (All studies– homogeneous network including inconsistencies)  OR [95% CrI] | Study pool 5 (consistent network)  OR [95% CrI] |
| Paroxetine | Sertraline vs. paroxetine |  |  | 0.59 [0.33; 0.98] | 0.28 [0.13; 0.54] |
|  | Fluvoxamine vs. paroxetine |  |  | 0.68 [0.29; 1.35] | 0.57 [0.27; 1.05] |
|  | TCAs vs. paroxetine |  |  | 1.06 [0.65; 1.66] | 0.87 [0.54; 1.42] |
|  | Agomelatine vs. paroxetine | 1 | 0.97 [0.31; 3.09] | 0.38 [0.20; 0.65] | 0.34 [0.19; 0.56] |
|  | Trazodone vs. paroxetine |  |  | 1.10 [0.48; 2.20] | 0.97 [0.44; 1.81] |
| Sertraline | Fluvoxamine vs. sertraline |  |  | 1.22 [0.46; 2.65] | 2.24 [0.84; 4.98] |
|  | TCAs vs. sertraline |  |  | 1.90 [0.99; 3.37] | 3.41 [1.52; 6.76] |
|  | Agomelatine vs. sertraline |  |  | 0.68 [0.31; 1.32] | 1.36 [0.56; 2.88] |
|  | Trazodone vs. sertraline |  |  | 1.98 [0.78; 4.30] | 3.84 [1.33; 8.77] |
| Fluvoxamine | TCAs vs. fluvoxamine |  |  | 1.80 [0.72; 3.84] | 1.71 [0.75; 3.46] |
|  | Agomelatine vs. fluvoxamine |  |  | 0.64 [0.23; 1.46] | 0.68 [0.27; 1.41] |
|  | Trazodone vs. fluvoxamine |  |  | 1.88 [0.58; 4.63] | 1.91 [0.66; 4.36] |
| TCAs | Agomelatine vs. TCAs |  |  | 0.37 [0.18; 0.67] | 0.41 [0.21; 0.69] |
|  | Trazodone vs. TCAs | 1 | 1.02 [0.24; 4.29] | 1.08 [0.46; 2.17] | 1.16 [0.51; 2.23] |
| Agomelatine | Trazodone vs. agomelatine |  |  | 3.16 [1.17; 6.95] | 2.97 [1.25; 6.03] |
| a: The model specification for this outcome is described in the main text of the present assessment and presented in Appendix H of the full report.  b: The exact value of the direct effect estimate is 1.003.  c: The exact value of the effect estimate of the MTC meta-analysis based on all studies is 1.0020.  CI: confidence interval, CrI: credible interval, DIC: deviance information criterion, MTC: mixed treatment comparison, OR: odds ratio, TCAs: tri- and tetracyclic antidepressants | | | | | |
