# Supplementary material for: Assumptions of Mixed Treatment Comparisons in Health Technology Assessments - Challenges and Possible Steps for Practical Application
Source: PLoS One. 2016 Aug 10;11(8):e0160712. doi: 10.1371/journal.pone.0160712 (PMC4979893; doi:10.1371/journal.pone.0160712)
Supplement: S4 Appendix — (DOCX) [file pone.0160712.s004.docx]

**S4 Appendix: Full list of studies**

Studies are listed by contrast in alphabetical order.

**AK130930 [bupropion vs. placebo]**

Modell JG, Rosenthal NE, Harriett AE, Krishen A, Asgharian A, Foster VJ et al. Seasonal affective disorder and its prevention by anticipatory treatment with bupropion XL. Biol Psychiatry 2005; 58(8): 658-667.

GlaxoSmithKline. A 7-month, multicenter, randomized, double-blind, placebo-controlled comparison of 150-300mg/day of extended-release bupropion hydrochloride (Wellbutrin XL) and placebo for the prevention of seasonal affective disorder in subjects with a history of seasonal affective disorder followed by an 8-week observational follow-up phase: clinical study report for study AK 130930 [unpublished]. 2004.

GlaxoSmithKline. A 7-month, multicenter, randomized, double-blind, placebo-controlled comparison of 150-300mg/day of extended-release bupropion hydrochloride (WELLBUTRIN XL) and placebo for the prevention of seasonal affective disorder in subjects with a history of seasonal affective disorder followed by an 8-week observational follow-up phase: study no. WELL AK130930 [online]. In: GlaxoSmithKline Clinical Study Register. 14.07.2007 [Access on: 25.08.2009]. URL: <http://www.gsk-clinicalstudyregister.com/files/pdf/20468.pdf>.

**AK130936 [bupropion vs. placebo]**

Modell JG, Rosenthal NE, Harriett AE, Krishen A, Asgharian A, Foster VJ et al. Seasonal affective disorder and its prevention by anticipatory treatment with bupropion XL. Biol Psychiatry 2005; 58(8): 658-667.

GlaxoSmithKline. A 7-month, multicenter, randomized, double-blind, placebo-controlled comparison of 150-300mg/day of extended-release bupropion hydrochloride (Wellbutrin XL) and placebo for the prevention of seasonal affective disorder in subjects with a history of seasonal affective disorder followed by an 8-week observational follow-up phase: clinical study report for study AK 130936 [unpublished]. 2004.

GlaxoSmithKline. A 7-month, multicenter, randomized, double-blind, placebo-controlled comparison of 150-300mg/day of extended-release bupropion hydrochloride (WELLBUTRIN XL) and placebo for the prevention of seasonal affective disorder in subjects with a history of seasonal affective disorder followed by an 8-week observational follow-up phase: study no. WELL AK130936 [online]. In: GlaxoSmithKline Clinical Study Register. 14.06.2007 [Access on: 25.08.2009]. URL: http://www.gsk-clinicalstudyregister.com/files/pdf/20471.pdf.

**AK130939 [venlafaxine vs. bupropion vs. placebo]**

GlaxoSmithKline. A multi-centre, randomised, double-blind, parallel-group, placebo- and active-controlled, flexible dose study evaluating the efficacy, safety and tolerability of extended-release bupropion hydrochloride (150 mg – 300 mg once daily), extended-release venlafaxine hydrochloride (75 mg – 150 mg once daily) and placebo in subjects with major depressive disorder: study AK130939 [Online]. In: GlaxoSmithKline Clinical Trial Register. 06.02.2006 [Access on 22.01.2008]. URL: http://ctr.gsk.co.uk/Summary/bupropion/III_AK130939.pdf.

GlaxoSmithKline. A multi-centre, randomised, double-blind, parallel-group, placebo- and active-controlled, flexible dose study evaluating the efficacy, safety and tolerability of extended-release bupropion hydrochloride (150 mg – 300 mg once daily), extended-release venlafaxine hydrochloride (75 mg – 150 mg once daily) and placebo in subjects with major depressive disorder: study AK130939 [unpublished]. 2005.

**AK130940 [bupropion vs. placebo]**

Hewett K, Chrzanowski W, Jokinen R, Felgentreff R, Shrivastava R, Gee M et al. Double-blind, placebo-controlled evaluation of extended-release bupropion in elderly patients with major depressive disorder. J Psychopharmacol 22.01.2009 [Epub ahead of print]

GlaxoSmithKline. A multi-centre, randomised, double-blind, parallel-group, placebo-controlled, flexible dose study to evaluate the efficacy, safety and tolerability of extended-release bupropion hydrochloride (150mg-300mg once daily) in elderly subjects with major depressive disorder: study AK 130940; clinical study report [unpublished]. 2006.

GlaxoSmithKline. A multi-centre, randomised, double-blind, parallel-group, placebo-controlled, flexible dose study to evaluate the efficacy, safety and tolerability of extended-release bupropion hydrochloride (150mg-300mg once daily) in elderly subjects with major depressive disorder: study no. AK 130940 [online]. In: GlaxoSmithKline Clinical Study Register. 16.05.2006 [Access on: 25.08.2009]. URL: <http://www.gsk-clinicalstudyregister.com/files/pdf/20472.pdf>.

**Allard 2004 [venlafaxine vs. citalopram]**

Allard P, Gram L, Timdahl K, Behnke K, Hanson M, Sogaard J. Efficacy and tolerability of venlafaxine in geriatric outpatients with major depression: a double-blind, randomised 6-month comparative trial with citalopram. Int J Geriatr Psychiatry 2004; 19(12): 1123-1130.

Wyeth Pharma GmbH. A randomised double-blind comparison of efficacy and tolerability of venlafaxine vs. Citalopram in depressed geriatric outpatients: study 4229-SW [unpublished].

**Alves 1999 [venlafaxine vs. fluoxetine]**

Alves C, Cachola I, Brandao J. Efficacy and tolerability of venlafaxine and fluoxetine in outpatients with major depression. Primary Care Psychiatry 1999; 5(2): 57-63.

**Amini 2005 [mirtazapine vs. fluoxetine]**

Amini H, Aghayan S, Jalili SA, Akhondzadeh S, Yahyazadeh O, Pakravan-Nejad M. Comparison of mirtazapine and fluoxetine in the treatment of major depressive disorder: a double-blind, randomized trial. J Clin Pharm Ther 2005; 30(2): 133-138.

**Berlanga 2006 [reboxetine vs. citalopram]**

Berlanga C, Flores-Ramos M. Different gender response to serotonergic and noradrenergic antidepressants: a comparative study of the efficacy of citalopram and reboxetine. J Affect Disord 2006; 95(1-3): 119-123.

**Benkert 1996 [venlafaxine vs. imipramine]**

Benkert O, Grunder G, Wetzel H, Hackett D. A randomized, double-blind comparison of a rapidly escalating dose of venlafaxine and imipramine in inpatients with major depression and melancholia. J Psychiatr Res 1996; 30(6): 441-451.

Wyeth Pharma GmbH. Randomized double-blind, comparative study of rapidly escalating doses of venlafaxine and imipramine in inpatients with major depression and melancholia: study 0600A-338-GE-CSR-44017 [unpublished].

**Benkert 2006 (C-1763) [venlafaxine vs. mirtazapine]**

Benkert O, Szegedi A, Philipp M, Kohnen R, Heinrich C, Heukels A et al. Mirtazapine orally disintegrating tablets versus venlafaxine extended release: a double-blind, randomized multicenter trial comparing the onset of antidepressant response in patients with major depressive disorder. J Clin Psychopharmacol 2006; 26(1): 75-78.

**Bielski 2004 [venlafaxine vs. escitalopram]**

Bielski RJ, Ventura D, Chang CC. A double-blind comparison of escitalopram and venlafaxine extended release in the treatment of major depressive disorder. J Clin Psychiatry 2004; 65(9): 1190-1196.

**Brannan 2005 (F1J-MC-HMCB) [duloxetine vs. placebo]**

Brannan SK, Mallinckrodt CH, Brown EB, Wohlreich MM, Watkin JG, Schatzberg AF. Duloxetine 60 mg once-daily in the treatment of painful physical symptoms in patients with major depressive disorder. J Psychiatr Res 2005; 39(1): 43-53.

Eli Lilly and Company. Duloxetine once-daily dosing versus placebo in patients with major depression and pain: summary ID 6353; study F1J-US-HMCB [Online]. In: ClinicalStudyResults. 27.03.2006 [Access on: 01.02.2007]. URL: <http://www.clinicalstudyresults.org/documents/company-study_1038_0.pdf>.

Eli Lilly and Company. Duloxetine once-daily dosing versus placebo in patients with major depression and pain: study F1J-US-HMCB [unpublished]. 2003.

**Brecht 2007 (F1J-BI-HMDH) [duloxetine vs. placebo]**

Brecht S, Courtecuisse C, Debieuvre C, Croenlein J, Desaiah D, Raskin J et al. Efficacy and safety of duloxetine 60 mg once daily in the treatment of pain in patients with major depressive disorder and at least moderate pain of unknown etiology: a randomized controlled trial. J Clin Psychiatry 2007; 68(11): 1707-1716.

Eli Lilly and Company. A ten-week, randomized, double-blind study evaluating the efficacy of duloxetine 60 mg once daily versus placebo in outpatients with major depressive disorder and pain: study F1J-BI-HMDH; clinical study summary #8605 [Online]. In: ClinicalStudyResults. 04.06.2007 [Access on 05.03.2008]. URL: http://www.clinicalstudyresults.org/documents/company-study_3498_0.pdf.

Eli Lilly and Company. A ten-week, randomized, double-blind study evaluating the efficacy of duloxetine 60 mg once daily versus placebo in outpatients with major depressive disorder and pain (EU-Pain enriched study): study number 1208.10; study F1J-BI-HMDH [unpublished]. 2006.

**CL2-014 [agomelatine vs. paroxetine vs. placebo]**

European Medicines Agency. Valdoxan: European public assessment report; public assessment report [online]. 20.11.2008 [Access on: 04.08.2011]. URL: http://www.ema.europa.eu/docs/en_GB/document_library/EPAR_-_Public_assessment_report/human/000915/WC500046226.pdf.

**CL3-021 [agomelatine vs. placebo]**

European Medicines Agency. Valdoxan: European public assessment report; public assessment report [online]. 20.11.2008 [Access on: 04.08.2011]. URL: http://www.ema.europa.eu/docs/en_GB/document_library/EPAR_-_Public_assessment_report/human/000915/WC500046226.pdf.

**CL3-022 [agomelatine vs. fluoxetine vs. placebo]**

European Medicines Agency. Valdoxan: European public assessment report; public assessment report [online]. 20.11.2008 [Access on: 04.08.2011]. URL: http://www.ema.europa.eu/docs/en_GB/document_library/EPAR_-_Public_assessment_report/human/000915/WC500046226.pdf.

**CL3-023 [agomelatine vs. paroxetine vs. placebo]**

European Medicines Agency. Valdoxan: European public assessment report; public assessment report [online]. 20.11.2008 [Access on: 04.08.2011]. URL: http://www.ema.europa.eu/docs/en_GB/document_library/EPAR_-_Public_assessment_report/human/000915/WC500046226.pdf.

**CL3-024 [agomelatine vs. fluoxetine vs. placebo]**

European Medicines Agency. Valdoxan: European public assessment report; public assessment report [online]. 20.11.2008 [Access on: 04.08.2011]. URL: http://www.ema.europa.eu/docs/en_GB/document_library/EPAR_-_Public_assessment_report/human/000915/WC500046226.pdf.

**CL3-026 [agomelatine vs. placebo]**

European Medicines Agency. Valdoxan: European public assessment report; public assessment report [online]. 20.11.2008 [Access on: 04.08.2011]. URL: http://www.ema.europa.eu/docs/en_GB/document_library/EPAR_-_Public_assessment_report/human/000915/WC500046226.pdf.

**CL3-041 [agomelatine vs. placebo]**

European Medicines Agency. Valdoxan: European public assessment report; public assessment report [online]. 20.11.2008 [Access on: 04.08.2011]. URL: http://www.ema.europa.eu/docs/en_GB/document_library/EPAR_-_Public_assessment_report/human/000915/WC500046226.pdf.

**CL3-042 [agomelatine vs. placebo]**

European Medicines Agency. Valdoxan: European public assessment report; public assessment report [online]. 20.11.2008 [Access on: 04.08.2011]. URL: http://www.ema.europa.eu/docs/en_GB/document_library/EPAR_-_Public_assessment_report/human/000915/WC500046226.pdf.

**CL3-043 [agomelatine vs. placebo]**

European Medicines Agency. Valdoxan: European public assessment report; public assessment report [online]. 20.11.2008 [Access on: 04.08.2011]. URL: http://www.ema.europa.eu/docs/en_GB/document_library/EPAR_-_Public_assessment_report/human/000915/WC500046226.pdf.

**Clerc 1994 [venlafaxine vs. fluoxetine]**

Clerc GE, Ruimy P, Verdeau-Pailles J. A double-blind comparison of venlafaxine and fluoxetine in patients hospitalized for major depression and melancholia. Int Clin Psychopharmacol 1994; 9(3): 139-143.

Wyeth Pharma GmbH. A randomized, double-blind, parallel group comparison of venlafaxine and fluoxetine in inpatients with major depression and melancholia: study 0600A-340-FR-GMR-21339 [unpublished].

**Corya 2006 [venlafaxine vs. fluoxetine]**

Corya SA, Williamson D, Sanger TM, Briggs SD, Case M, Tollefson G. A randomized, double-blind comparison of olanzapine/fluoxetine combination, olanzapine, fluoxetine, and venlafaxine in treatment-resistant depression. Depress Anxiety 2006; 23(6): 364-372.

Eli Lilly and Company. Olanzapine plus fluoxetine combination therapy in treatment-resistant depression: a dose ranging study; study F1D-MC-HGIE; clinical study summary #3641 [Online]. In: ClinicalStudyResults. 21.06.2006 [Access on 22.01.2008]. URL: http://www.clinicalstudyresults.org/documents/company-study_2107_0.pdf.

Eli Lilly and Company. Olanzapine plus fluoxetine combination therapy in treatment-resistant depression: a dose ranging study: study F1D-MC-HGIE [unpublished]. 2002.

**Costa e Silva 1998 [venlafaxine vs. fluoxetine]**

Costa E, Silva J. Randomized, double-blind comparison of venlafaxine and fluoxetine in outpatients with major depression. J Clin Psychiatry 1998; 59(7): 352-357.

**Cunningham 1994 [venlafaxine vs. trazodon vs. placebo]**

Cunningham LA, Borison RL, Carman JS, Chouinard G, Crowder JE, Diamond BI et al. A comparison of venlafaxine, trazodone, and placebo in major depression. J Clin Psychopharmacol 1994; 14(2): 99-106.

Wyeth Pharma GmbH. Randomized double-blind comparison of venlafaxine, trazodone and placebo capsules in outpatients with major depression: study 0600A-302-US-CA-GMR-20334 [unpublished].

**Cunningham 1997 [venlafaxine vs. placebo]**

Cunningham LA. Once-daily venlafaxine extended release (XR) and venlafaxine immediate release (IR) in outpatients with major depression. Ann Clin Psychiatry 1997; 9(3): 157-164.

Wyeth Pharma GmbH. A double-blind, placebo-controlled study of venlafaxine ER in outpatients with major depression: study 0600B-208-US-GMR-26165 [unpublished].

**Detke 2002a (F1J-MC-HMBH-B) [duloxetine vs. placebo]**

Detke MJ, Lu Y, Goldstein DJ, McNamara RK, Demitrack MA. Duloxetine 60 mg once daily dosing versus placebo in the acute treatment of major depression. J Psychiatr Res 2002; 36(6): 383-390.

Eli Lilly and Company. Duloxetine once-daily dosing versus placebo in the acute treatment of major depression: study F1J-MC-HMBH, study group B; clinical study summary #4689 [Online]. In: ClinicalStudyResults. 23.11.2004 [Access on 30.01.2006]. URL: http://www.clinicalstudyresults.org/documents/company-study_142_0.pdf.

Eli Lilly and Company. Duloxetine once-daily dosing versus placebo in the acute treatment of major depression: study F1J-MC-HMBH, study group B [unpublished]. 2001.

**Detke 2002b (F1J-MC-HMBH-A) [duloxetine vs. placebo]**

Detke MJ, Lu Y, Goldstein DJ, Hayes JR, Demitrack MA. Duloxetine, 60 mg once daily, for major depressive disorder: a randomized double-blind placebo-controlled trial. J Clin Psychiatry 2002; 63(4): 308-315.

Eli Lilly and Company. Duloxetine once-daily dosing versus placebo in the acute treatment of major depression: study F1J-MC-HMBH, study group A; clinical study summary #4689 [Online]. In: ClinicalStudyResults. 15.11.2004 [Access on 30.01.2006]. URL: http://www.clinicalstudyresults.org/documents/company-study_141_0.pdf.

Eli Lilly and Company. Duloxetine once-daily dosing versus placebo in the acute treatment of major depression: study F1J-MC-HMBH, study group A [unpublished]. 2001.

**Detke 2004 (F1J-MC-HMAY-A) [duloxetine vs. paroxetine vs. placebo]**

Detke MJ, Wiltse CG, Mallinckrodt CH, McNamara RK, Demitrack MA, Bitter I. Duloxetine in the acute and long-term treatment of major depressive disorder: a placebo- and paroxetine-controlled trial. Eur Neuropsychopharmacol 2004; 14(6): 457-470.

Eli Lilly and Company. Duloxetine versus placebo and paroxetine in the treatment of major depression: study F1J-MC-HMAY, study group A; clinical study summary #4298 [Online]. In: ClinicalStudyResults. 23.06.2005 [Access on 30.01.2006]. URL: http://www.clinicalstudyresults.org/documents/company-study_887_0.pdf.

Eli Lilly and Company. Duloxetine versus placebo and paroxetine in the treatment of major depression: study F1J-MC-HMAY, study group A [unpublished]. 2003.

**De Vasconcelos Cunha 2007 [venlafaxine vs. placebo]**

De Vasconcelos Cunha UG, Lopes Rocha F, Avila de Melo R, Alves Valle E, De Souza Neto JJ, Mendes Brega R et al. A placebo-controlled double-blind randomized study of venlafaxine in the treatment of depression in dementia. Dement Geriatr Cogn Disord 2007; 24(1): 36-41.

**Dierick 1996 [venlafaxine vs. fluoxetine]**

Dierick M, Ravizza L, Realini R, Martin A. A double-blind comparison of venlafaxine and fluoxetine for treatment of major depression in outpatients. Prog Neuropsychopharmacol Biol Psychiatry 1996; 20(1): 57-71.

Wyeth Pharma GmbH. A randomized, double-blind comparison of the efficacy and safety of venlafaxine versus fluoxetine in depressed outpatients: study 600A1-348-FR-GMR-23765 [unpublished].

**E-1559 [mirtazapine vs. paroxetine]**

Benkert O, Szegedi A, Kohnen R. Mirtazapine compared with paroxetine in major depression. J Clin Psychiatry 2000; 61(9): 656-663.

Organon. Single-centre, double-blind, randomized, paroxetine controlled efficacy and safety study with Remergil (Org 3770) in depressed patients: study E-1559; integrated clinical and statistical study report; final version 3.0 [unpublished]. 1999.

Szegedi A, Muller MJ, Anghelescu I, Klawe C, Kohnen R, Benkert O. Early improvement under mirtazapine and paroxetine predicts later stable response and remission with high sensitivity in patients with major depression. J Clin Psychiatry 2003; 64(4): 413-420.

Szegedi A, Rujescu D, Tadic A, Muller MJ, Kohnen R, Stassen HH et al. The catechol-O-methyltransferase Val108/158Met polymorphism affects short-term treatment response to mirtazapine, but not to paroxetine in major depression. Pharmacogenomics J 2005; 5(1): 49-53.

Tadi A, Müller MJ, Rujescu D, Kohnen R, Stassen HH, Dahmen N et al. The MAOA T941G polymorphism and short-term treatment response to mirtazapine and paroxetine in major depression. Am J Med Genet B Neuropsychiatr Genet 2007; 144B(3): 325-331.

Tadi A, Rujescu D, Müller MJ, Kohnen R, Stassen HH, Dahmen N et al. A monoamine oxidase B gene variant and short-term antidepressant treatment response. Prog Neuropsychopharmacol Biol Psychiatry 2007; 31(7): 1370-1377.

Tadic A, Rujescu D, Müller MJ, Kohnen R, Stassen HH, Szegedi A et al. Association analysis between variants of the interleukin-1beta and the interleukin-1 receptor antagonist gene and antidepressant treatment response in major depression. Neuropsychiatr Dis Treat 2008; 4(1): 269-276.

**E-1569 [mirtazapine vs. paroxetine]**

Organon. A multicentre, randomised, double-blind group comparative study comparing the tolerability of six weeks treatment with Remeron (Org 3770) and paroxetine in depressed patients in general practice: clinical report on protocol E-1569 [unpublished]. 1998.

**E-1639 [mirtazapine vs. paroxetine]**

Organon. A single-center, randomized, double blind group comparative study on the therapeutic effects of six weeks treatment with mirtazapine, paroxetine and their combination in 60 patients with major depression: clinical trial report on protocol E-1639, without bioanalysis and PET-scan analysis [unpublished]. 2002.

**E-1690 [mirtazapine vs. sertaline]**

Behnke K, Sogaard J, Martin S, Bauml J, Ravindran AV, Agren H et al. Mirtazapine orally disintegrating tablet versus sertraline: a prospective onset of action study. J Clin Psychopharmacol 2003; 23(4): 358-364.

Organon. A multicenter, double blind randomized sertraline-controlled efficacy and safety study with mirtazapine (Remeron) in subjects with a major depressive episode (according to DSM-IV criteria): clinical trial report on protocol E-1690 [unpublished]. 2002.

**E-1721 / Wade 2003 [mirtazapine vs. paroxetine]**

Wade A, Crawford GM, Angus M, Wilson R, Hamilton L. A randomized, double-blind, 24-week study comparing the efficacy and tolerability of mirtazapine and paroxetine in depressed patients in primary care. Int Clin Psychopharmacol 2003; 18(3): 133-141.

Romeo R, Patel A, Knapp M, Thomas C. The cost-effectiveness of mirtazapine versus paroxetine in treating people with depression in primary care. Int Clin Psychopharmacol 2004; 19(3): 125-134.

Akzo Nobel. A 24 week double-blind, randomised, controlled trial comparint the efficacy, tolerability and health economic aspects of mirtazapine and paroxetine in depressed patients presenting to their general practioners in the United Kingdom: clinical trial report on protocol E1721 [unpublished]. 2003.

**F1J-MC-HMBU [duloxetine vs. venlafaxine]**

Eli Lilly and Company. Duloxetine versus venlafaxine extended release in the treatment of major depressive disorder: study F1J-MC-HMBU; clinical study summary [Online]. In: Lilly Clinical Trial Registry. 27.02.2008 [Access on 20.06.2008]. URL: <http://www.clinicalstudyresults.org/documents/company-study_3107_0.pdf>.

Eli Lilly and Company. Duloxetine versus venlafaxine extended release in the treatment of major depressive disorder: study F1J-MC-HMBU [unpublished]. 2004.

**F1J-MC-HMCQ [duloxetine vs. venlafaxine]**

Eli Lilly and Company. Duloxetine versus venlafaxine extended release in the treatment of major depressive disorder: study F1J-MC-HMCQ; clinical study summary [Online]. In: Lilly Clinical Trial Registry. 14.03.2008 [Access on 20.06.2008]. URL: http://www.clinicalstudyresults.org/documents/company-study_3108_0.pdf.

Eli Lilly and Company. Duloxetine versus venlafaxine extended release in the treatment of major depressive disorder: study F1J-MC-HMCQ [unpublished]. 2004

**Gentil 2000 [venlafaxine vs. amitryptiline]**

Gentil V, Benedictis E, Kerr-Correa F, Moreno R, Busnello ED, De Campos JA et al. Double-blind comparison of venlafaxine and amitriptyline in outpatients with major depression with or without melancholia. J Psychopharmacol 2000; 14(1): 61-66.

**GMA-016-CSR [venlafaxine vs. fluoxetine vs. placebo]**

Wyeth Pharma GmbH. Double-blind, placebo-controlled study of venlafaxine and fluoxetine in patients with major depression and melancholia: study GMA-016-CSR [unpublished].

**Goldstein 2002 (F1J-MC-HMAQ-A) [duloxetine vs. fluoxetine vs. placebo]**

Goldstein DJ, Mallinckrodt C, Lu Y, Demitrack MA. Duloxetine in the treatment of major depressive disorder: a double-blind clinical trial. J Clin Psychiatry 2002; 63(3): 225-231.

Eli Lilly and Company. Duloxetine versus placebo in the treatment of major depression: study F1J-MC-HMAQ, study group A; clinical study synopsis #3327 [Online]. In: ClinicalStudyResults. 16.11.2004 [Access on 30.01.2006]. URL: http://www.clinicalstudyresults.org/documents/company-study_137_0.pdf.

Eli Lilly and Company. Duloxetine versus placebo in the treatment of major depression: study F1J-MC-HMAQ, study group A [unpublished]. 2001.

**Goldstein 2004 (F1J-MC-HMAT-B) [duloxetine vs. paroxetine vs. placebo]**

Goldstein DJ, Lu Y, Detke MJ, Wiltse C, Mallinckrodt C, Demitrack MA. Duloxetine in the treatment of depression: a double-blind placebo-controlled comparison with paroxetine. J Clin Psychopharmacol 2004; 24(4): 389-399.

Eli Lilly and Company. Duloxetine versus placebo and paroxetine in the acute treatment of major depression: study F1J-MC-HMAT, study group B; clinical study summary #4091 [Online]. In: ClinicalStudyResults. 16.11.2004 [Access on 30.01.2006]. URL: http://www.clinicalstudyresults.org/documents/company-study_139_0.pdf.

Eli Lilly and Company. Duloxetine versus placebo and paroxetine in the acute treatment of major depression: study F1J-MC-HMAT, study group B [unpublished]. 2001.

**HMAT-A (F1J-MC-HMAT-A) [duloxetine vs. paroxetine vs. placebo]**

Eli Lilly and Company. Duloxetine versus placebo and paroxetine in the acute treatment of major depression: study F1J-MC-HMAT, study group A; clinical study summary #4091 [Online]. In: ClinicalStudyResults. 16.11.2006 [Access on 30.01.2006]. URL: http://www.clinicalstudyresults.org/documents/company-study_170_0.pdf.

Eli Lilly and Company. Duloxetine versus placebo and paroxetine in the acute treatment of major depression: study F1J-MC-HMAT, study group A [unpublished]. 2001.

**HMAQ-B (F1J-MC-HMAQ-B) [duloxetine vs. fluoxetine vs. placebo]**

Eli Lilly and Company. Duloxetine versus placebo in the treatment of major depression: study F1J-MC-HMAQ, study group B; clinical study summary #3327 [Online]. In: ClinicalStudyResults. 16.11.2006 [Access on 30.01.2006]. URL: http://www.clinicalstudyresults.org/documents/company-study_138_0.pdf.

Eli Lilly and Company. Duloxetine versus placebo in the treatment of major depression: study F1J-MC-HMAQ, study group B [unpublished]. 2001.

**Honig 2007 [mirtazapine vs. placebo]**

Honig A, Kuyper AMG, Schene AH, Van Melle JP, De Jonge P, Tulner DM et al. Treatment of post-myocardial infarction depressive disorder: a randomized, placebo-controlled trial with mirtazapine. Psychosom Med 2007; 69(7): 606-613.

Van den Brink RHS, Van Melle JP, Honig A, Schene AH, Crijns HJGM, Lambert FPG et al. Treatment of depression after myocardial infarction and the effects on cardiac prognosis and quality of life: rationale and outline of the Myocardial INfarction and Depression-Intervention Trial (MIND-IT). Am Heart J 2002; 144(2): 219-225.

Schins A, Hamulyak K, Scharpe S, Lousberg R, Van Melle J, Crijns H et al. Whole blood serotonin and platelet activation in depressed post-myocardial infarction patients. Life Sci 2004; 76(6): 637-650.

De Jonge P, Honig A, Van Melle JP, Schene AH, Kuyper AMG, Tulner D et al. Nonresponse to treatment for depression following myocardial infarction: association with subsequent cardiac events.[see comment]. Am J Psychiatry 2007; 164(9): 1371-1378.

**Keller 2007a [venlafaxine vs. fluoxetine]**

Keller MB, Trivedi MH, Thase ME, Shelton RC, Kornstein SG, Nemeroff CB et al. The Prevention of Recurrent Episodes of Depression with Venlafaxine for Two Years (PREVENT) Study: outcomes from the acute and continuation phases. Biol Psychiatry 2007; 62(12): 1371-1379.

Kornstein SG. Beyond remission: rationale and design of the prevention of recurrent episodes of depression with venlafaxine for two years (PREVENT) study. CNS Spectrums 2006; 11(12 Suppl 15): 28-34.

Wyeth Pharma GmbH. A acute and continuation phase study of the comparative efficacy of venlafaxine ER (Effexor XR) and fluoxetine (PROZAC) in achieving and sustaining remission (wellness) in patients with recurrent unipolar major depression; followed by long term randomized, placebo-controlled maintenance treatment study in patients treated initially with venlafaxine ER: study 0600B-100469-GMA-CSR-64337 [unpublished].

**Keller 2007b / Kocsis 2007 [venlafaxine vs. placebo]**

Keller MB, Trivedi MH, Thase ME, Shelton RC, Kornstein SG, Nemeroff CB et al. The Prevention of Recurrent Episodes of Depression with Venlafaxine for Two Years (PREVENT) Study: outcomes from the 2-year and combined maintenance phases. J Clin Psychiatry 2007; 68(8): 1246-1256.

Kocsis JH, Thase ME, Trivedi MH, Shelton RC, Kornstein SG, Nemeroff CB et al. Prevention of recurrent episodes of depression with venlafaxine ER in a 1-year maintenance phase from the PREVENT study. J Clin Psychiatry 2007; 68(7): 1014-1023.

Kornstein SG. Beyond remission: rationale and design of the prevention of recurrent episodes of depression with venlafaxine for two years (PREVENT) study. CNS Spectrums 2006; 11(12 Suppl 15): 28-34.

Wyeth Pharma GmbH. A acute and continuation phase study of the comparative efficacy of venlafaxine ER (Effexor XR) and fluoxetine (PROZAC) in achieving and sustaining remission (wellness) in patients with recurrent unipolar major depression; followed by long term randomized, placebo-controlled maintenance treatment study in patients treated initially with venlafaxine ER: study 0600B-100469-GMA-CSR-64337 [unpublished].

**Kennedy 2008 [venlafaxine vs. agomelatine]**

Kennedy SH, Rizvi S, Fulton K, Rasmussen J. A double-blind comparison of sexual functioning, antidepressant efficacy, and tolerability between agomelatine and venlafaxine XR. J Clin Psychopharmacol 2008; 28(3): 329-333.

**Khan 1998 [venlafaxine vs. placebo]**

Khan A, Upton GV, Rudolph RL, Entsuah R, Leventer SM. The use of venlafaxine in the treatment of major depression and major depression associated with anxiety: a dose-response study. J Clin Psychopharmacol 1998; 18(1): 19-25.

Wyeth Pharma GmbH. Double-blind, placebo-controlled evaluation of three twice-daily doses of venlafaxine in patients with major depression: study 0600A1-342-US-GMR-24199 [unpublished].

**Khan 2007 [duloxetine vs. escitalopram]**

Khan A, Bose A, Alexopoulos GS, Gommoll C, Li D, Gandhi C. Double-blind comparison of escitalopram and duloxetine in the acute treatment of major depressive disorder. Clin Drug Investig 2007; 27(7): 481-492.

**Kok 2007 [venlafaxine vs. nortriptyline]**

Kok RM, Nolen WA, Heeren TJ. Venlafaxine versus nortriptyline in the treatment of elderly depressed inpatients: a randomised, double-blind, controlled trial. Int J Geriatr Psychiatry 2007; 22(12): 1247-1254.

Kok R, Nolen W, Heeren T. Cardiovascular changes associated with venlafaxine in the treatment of late-life depression. Am J Geriatr Psychiatry 2007; 15(8): 725.

**Lecrubier 1997 [venlafaxine vs. imipramine vs. placebo]**

Lecrubier Y, Bourin M, Moon CAL, Schifano F, Blanchard C, Danjou P et al. Efficacy of venlafaxine in depressive illness in general practice. Acta Psychiatr Scand 1997; 95(6): 485-493.

Wyeth Pharma GmbH. Randomized double-blind, comparative study of the safety and efficacy of venlafaxine, imipramine and placebo capsules in outpatients with mild to moderate depression: study 0600A1-346-UK-IT-FR-CSR-39785 [unpublished].

**Lee 2007 (F1J-AA-HMCV) [duloxetine vs. paroxetine]**

Lee P, Shu L, Xu X, Wang CY, Lee MS, Liu CY et al. Once-daily duloxetine 60 mg in the treatment of major depressive disorder: multicenter, double-blind, randomized, paroxetine-controlled, non-inferiority trial in China, Korea, Taiwan and Brazil. Psychiatry Clin Neurosci 2007; 61(3): 295-307.

Eli Lilly and Company. Duloxetine versus paroxetine in the acute treatment of major depression: study F1J-AA-HMCV; clinical study summary #6937 [Online]. In: ClinicalStudyResults. 10.08.2006 [Access on 02.05.2007]. URL: http://www.clinicalstudyresults.org/documents/company-study_2402_0.pdf.

Eli Lilly and Company. Duloxetine versus paroxetine in the acute treatment of major depression: study F1J-AA-HMCV [unpublished]. 2006.

**Lemoine 2007 [venlafaxine vs. agomelatine]**

Lemoine P, Guilleminault C, Alvarez E. Improvement in subjective sleep in major depressive disorder with a novel antidepressant, agomelatine: randomized, double-blind comparison with venlafaxine. J Clin Psychiatry 2007; 68(11): 1723-1732.

**Mahapatra 1997 [venlafaxine vs. dothiepin]**

Mahapatra SN, Hackett D. A randomised, double-blind, parallel-group comparison of venlafaxine and dothiepin in geriatric patients with major depression. Int J Clin Pract 1997; 51(4): 209-213.

Wyeth Pharma GmbH. A double-blind, parallel-group of venlafaxine and dothiepin capsules in geriatric patients with major depression: study 0600A-316-EU-GMR-20003 [unpublished].

**McPartlin 1998 [venlafaxine vs. paroxetine]**

McPartlin GM, Reynolds A, Anderson C, Casoy J. A comparison of once-daily venlafaxine XR and paroxetine in depressed outpatients treated in general practice. Primary Care Psychiatry 1998; 4(3): 127-132.

**Mehtonen 2000 [venlafaxine vs. sertaline]**

Mehtonen OP, Sogaard J, Roponen P, Behnke K. Randomized, double-blind comparison of venlafaxine and sertraline in outpatients with major depressive disorder. J Clin Psychiatry 2000; 61(2): 95-100.

Wyeth Pharma GmbH. Randomized double-blind comparison of the safety and efficacy of venlafaxine versus sertraline in the treatment of inpatients and outpatients with major depression: study 600A-631-DE-FI [unpublished].

**Mendels 1993 [venlafaxine vs. placebo]**

Mendels J, Johnston R, Mattes J, Riesenberg R. Efficacy and safety of b.i.d. doses of venlafaxine in a dose-response study. Psychopharmacol Bull 1993; 29(2): 169-174.

Wyeth Pharma GmbH. Double-blind, placebo-controlled, parallel-group dosage-determination study of low doses of venlafaxine in depressed patients: study 0600A-313-US-GMR-19056 [unpublished].

**Montgomery 2004a [venlafaxine vs. placebo]**

Montgomery SA, Entsuah R, Hackett D, Kunz NR, Rudolph RL, Study G. Venlafaxine versus placebo in the preventive treatment of recurrent major depression. J Clin Psychiatry 2004; 65(3): 328-336.

Wyeth Pharma GmbH. Six-months, open-label evaluation of the safety and efficacy of venlafaxine followed by a randomized, double-blind, placebo-controlled, one-year evaluation of venlafaxine in the prophylactic treatment of recurrent major depression: study 0600A1-335-EU-US-CSR-32657 [unpublished].

**Montgomery 2004b [venlafaxine vs. escitalopram]**

Montgomery SA, Huusom AKT, Bothmer J. A randomised study comparing escitalopram with venlafaxine XR in primary care patients with major depressive disorder. Neuropsychobiology 2004; 50(1): 57-64.

**Nemeroff 2007 [venlafaxine vs. fluoxetine vs. placebo]**

Nemeroff CB, Thase ME, Group ES. A double-blind, placebo-controlled comparison of venlafaxine and fluoxetine treatment in depressed outpatients. J Psychiatr Res 2007; 41(3-4): 351-359.

Wyeth Pharma GmbH. Double-blind, placebo-controlled study of venlafaxine and fluoxetine in outpatients with major depression: study GMA-014-CSR [unpublished].

**Nierenberg 2007 (F1J-US-HMCR) [duloxetine vs. escitalopram vs. placebo]**

Nierenberg AA, Greist JH, Mallinckrodt CH, Prakash A, Sambunaris A, Tollefson GD et al. Duloxetine versus escitalopram and placebo in the treatment of patients with major depressive disorder: onset of antidepressant action, a non-inferiority study. Curr Med Res Opin 2007; 23(2): 401-416.

Eli Lilly and Company. Duloxetine versus escitalopram and placebo in the treatment of patients with major depression: study F1J-US-HMCR; clinical study summary #7978 [Online]. In: ClinicalStudyResults. 12.02.2007 [Access on 13.04.2007]. URL: http://www.clinicalstudyresults.org/documents/company-study_2182_0.pdf.

Eli Lilly and Company. Duloxetine versus escitalopram and placebo in the treatment of patients with major depression: study F1J-US-HMCR [unpublished]. 2005.

**Perahia 2006a (F1J-MC-HMBC)**

Perahia DG, Gilaberte I, Wang F, Wiltse CG, Huckins SA, Clemens JW et al. Duloxetine in the prevention of relapse of major depressive disorder: double-blind placebo-controlled study. Br J Psychiatry 2006; 188(4): 346-353.

Eli Lilly and Company. Duloxetine versus placebo in the prevention of relapse of major depressive disorder: study F1J-MC-HMBC; clinical study summary #4445 [Online]. In: ClinicalStudyResults. 12.07.2006 [Access on 29.11.2006]. URL: http://www.clinicalstudyresults.org/documents/company-study_1504_0.pdf.

Eli Lilly and Company. Duloxetine versus placebo in the prevention of relapse of major depressive disorder: study F1J-MC-HMBC [unpublished]. 2003.

**Perahia 2006b (F1J-MC-HMAY-B) [duloxetine vs. paroxetine vs. placebo]**

Perahia DGS, Wang F, Mallinckrodt CH, Walker DJ, Detke MJ. Duloxetine in the treatment of major depressive disorder: a placebo- and paroxetine-controlled trial. Eur Psychiatry 2006; 21(6): 367-378.

Eli Lilly and Company. Duloxetine versus placebo and paroxetine in the treatment of major depression: study F1J-MC-HMAY, study group B; clinical study summary #4298 [Online]. In: ClinicalStudyResults. 27.07.2006 [Access on 16.05.2006]. URL: http://www.clinicalstudyresults.org/documents/company-study_1618_0.pdf.

Eli Lilly and Company. Duloxetine versus placebo and paroxetine in the treatment of major depression: study F1J-MC-HMAY, study group B [unpublished]. 2003.

**Raskin 2007 (F1J-MC-HMBV) [duloxetine vs. placebo]**

Raskin J, Wiltse CG, Siegal A, Sheikh J, Xu J, Dinkel JJ et al. Efficacy of duloxetine on cognition, depression, and pain in elderly patients with major depressive disorder: an 8-week, double-blind, placebo-controlled trial. Am J Psychiatry 2007; 164(6): 900-909.

Wise TN, Wiltse CG, Iosifescu DV, Sheridan M, Xu JY, Raskin J. The safety and tolerability of duloxetine in depressed elderly patients with and without medical comorbidity. Int J Clin Pract 2007; 61(8): 1283-1293.

Eli Lilly and Company. Duloxetine versus placebo in the treatment of elderly patients with major depressive disorder: study F1J-MC-HMBV; clinical study summary #6091 [Online]. In: ClinicalStudyResults. 02.08.2005 [Access on 27.07.2007]. URL: http://www.clinicalstudyresults.org/documents/company-study_2651_0.pdf.

Eli Lilly and Company. Duloxetine versus placebo in the treatment of elderly patients with major depressive disorder: study F1J-MC-HMBV [unpublished]. 2004.

**Rudolph 1998 [venlafaxine vs. placebo]**

Rudolph RL, Fabre LF, Feighner JP, Rickels K, Entsuah R, Derivan AT. A randomized, placebo-controlled, dose-response trial of venlafaxine hydrochloride in the treatment of major depression. J Clin Psychiatry 1998; 59(3): 116-122.

Wyeth Pharma GmbH. Double-blind, placebo-controlled, parallel-group dosgae-determination study of venlafaxine tablets in depressed patients: study 0600A-203-US-GMR-16798 [unpublished].

**Rudolph 1999 [venlafaxine vs. fluoxetine vs. placebo]**

Rudolph RL, Feiger AD. A double-blind, randomized, placebo-controlled trial of once-daily venlafaxine extended release (XR) and fluoxetine for the treatment of depression. J Affect Disord 1999; 56(2-3): 171-181.

Wyeth Pharma GmbH. A double-blind, randomized, placebo controlled, trial of once-daily venlafaxine ER and fluoxetine for the treatment of depression: study 0600A1-211-US-GMR-33319 [unpublished].

**Samuelian 1998 [venlafaxine vs. clomipramine]**

Samuelian JC, Hackett D. A randomized, double-blind, parallel-group comparison of venlafaxine and clomipramine in outpatients with major depression. J Psychopharmacol 1998; 12(3): 273-278.

Wyeth Pharma GmbH. A randomized, double-blind, parallel group comparison of venlafaxine and clomipramine capsules in outpatients with major depression: study 0600A-315-GMR-18104 [unpublished].

**Sauer 2003 [venlafaxine vs. amitriptyline]**

Sauer H, Huppertz-Helmhold S, Dierkes W. Efficacy and safety of venlafaxine ER vs. amitriptyline ER in patients with major depression of moderate severity. Pharmacopsychiatry 2003; 36(5): 169-175.

Wyeth Pharma GmbH. Doppelblinde, randomisierte Phase-III-Studie zum Vergleich der Wirksamkeit und Verträglichkeit von Venlafaxin ER versus Amitriptylin ER bei Patienten mit einer Depression mittleren Schweregrades: Studie 0600B-100439-10-95-Venla-GE-1 [unpublished].

**Schatzberg 2006 [venlafaxine vs. fluoxetine vs. placebo]**

Schatzberg A, Roose S. A double-blind, placebo-controlled study of venlafaxine and fluoxetine in geriatric outpatients with major depression. Am J Geriatr Psychiatry 2006; 14(4): 361-370.

Wyeth Pharma GmbH. Double-blind, placebo-controlled study of venlafaxine and fluoxetine in geriatric outpatients with major depression: final report: study GMA-015-CSR [unpublished].

**Schweizer 1994 [venlafaxine vs. placebo]**

Schweizer E, Feighner J, Mandos LA, Rickels K. Comparison of venlafaxine and imipramine in the acute treatment of major depression in outpatients. J Clin Psychiatry 1994; 55(3): 104-108.

Wyeth Pharma GmbH. Randomized double-blind comaprison of venlafaxine, imipramine and placebo capsules in outpatients with major depression: study 0600A-301-US-301-EXT-US-GMR-18062 [unpublished].

**SCT-MD-35 [bupropion vs. placebo]**

Forest Laboratories. Fixed-dose comparison of escitalopram combination in adult patients with major depressive disorder: study SCT-MD-35 [online]. In: Forest Laboratories Clinical Trial Registry. 2007 [Access on: 25.08.2009]. URL: <http://www.forestclinicaltrials.com/CTR/CTRController/CTRViewPdf?_file_id=scsr/SCSR_SCT-MD-35_final.pdf>.

Forest Research Institute. Fixed-dose comparison of escitalopram combination in adult patients with major depressive disorder: study report; study no. SCT-MD-35 [unpublished]. 2007.

**Shelton 2006 [venlafaxine vs. sertraline]**

Shelton RC, Haman KL, Rapaport MH, Kiev A, Smith WT, Hirschfeld RMA et al. A randomized, double-blind, active-control study of sertraline versus venlafaxine XR in major depressive disorder. J Clin Psychiatry 2006; 67(11): 1674-1681.

**Silverstone 1999 [venlafaxine vs. fluoxetine vs. placebo]**

Silverstone PH, Ravindran A. Once-daily venlafaxine extended release (XR) compared with fluoxetine in outpatients with depression and anxiety. J Clin Psychiatry 1999; 60(1): 22-28.

Wyeth Pharma GmbH. A randomised, double-blind, placebo-controlled study of the efficacy and safety of venlafaxine extended release versus fluoxetine in depressed outpatients with concomitant anxiety: study 0600B1-360-CA-GMR-30410 [unpublished].

**Simon 2004 [venlafaxine vs. placebo]**

Simon JS, Aguiar LM, Kunz NR, Lei D. Extended-release venlafaxine in relapse prevention for patients with major depressive disorder. J Psychiatr Res 2004; 38(3): 249-257.

Wyeth Pharma GmbH. An evaluation of venlafaxine ER in the prevention of relapse in outpatients with major depression: study 0600B1-370-US-12-9.1-CSR-36951 [unpublished].

**Sir 2005 [velnafaxine vs. sertaline]**

Sir A, D'Souza RF, Uguz S, George T, Vahip S, Hopwood M et al. Randomized trial of sertraline versus venlafaxine XR in major depression: efficacy and discontinuation symptoms. J Clin Psychiatry 2005; 66(10): 1312-1320.

Pfizer Inc. A multicenter randomized, double-blind, parallel-group study of sertraline versus venlafaxine XR in the acute treatment of outpatients with major depressive disorder: protocol A0501066 [Online]. In: ClinicalStudyResults. 15.05.2005 [Access on 22.01.2008]. URL: http://www.clinicalstudyresults.org/documents/company-study_1991_0.pdf.

**Smeraldi 1998 [venlafaxine vs. clomipramine vs. trazodon]**

Smeraldi E, Rizzo F, Crespi G. Double-blind, randomized study of venlafaxine, clomipramine and trazodone in geriatric patients with major depression. Primary Care Psychiatry 1998; 4(4): 189-195.

**TAI-VENXR002-SDC-TRIAL-4156 [venlafaxine vs. fluoxetine]**

Wyeth Pharma GmbH. A double-Blind, randomized 8-week study of the safety and efficacy of venlafaxine extended release (ER) compared to fluoxetine in patients with moderate to severe depression: study TAI-VENXR002-SDC-TRIAL-4156 [unpublished].

**Thase 1997 [venlafaxine vs. placebo]**

Thase ME. Efficacy and tolerability of once-daily venlafaxine extended release (XR) in outpatients with major depression. J Clin Psychiatry 1997; 58(9): 393-398.

Wyeth Pharma GmbH. Double-blind, placebo-controlled study of venlafaxine ER in outpatients with major depression: study 0600B-209-US-GMR-27258 [unpublished].

**Trick 2004 [venlafaxine vs. dothiepin]**

Trick L, Stanley N, Rigney U, Hindmarch I. A double-blind, randomized, 26-week study comparing the cognitive and psychomotor effects and efficacy of 75 mg (37.5 mg b.i.d.) venlafaxine and 75 mg (25 mg mane, 50 mg nocte) dothiepin in elderly patients with moderate major depression being treated in general practice. J Psychopharmacol 2004; 18(2):205-214.

**Tylee 1997 [venlafaxine vs. fluoxetine]**

Tylee A, Beaumont G, Bowden MW, Reynolds A. A double-blind, randomized, 12-week comparison study of the safety and efficacy of venlafaxine and fluoxetine in moderate to severe major depression in general practice. Primary Care Psychiatry 1997; 3(1): 51-58.

**Tzanakaki 2000 [venlafaxine vs. fluoxetine]**

Tzanakaki M, Guazzelli M, Nimatoudis I, Zissis NP, Smeraldi E, Rizzo F. Increased remission rates with venlafaxine compared with fluoxetine in hospitalized patients with major depression and melancholia. Int Clin Psychopharmacol 2000; 15(1): 29-34.

**Wade 2007 [duloxetine vs. escitalopram]**

Wade A, Gembert K, Florea I. A comparative study of the efficacy of acute and continuation treatment with escitalopram versus duloxetine in patients with major depressive disorder. Curr Med Res Opin 2007; 23(7): 1605-1614.

**WELL100006 [bupropion vs. placebo]**

Modell JG, Rosenthal NE, Harriett AE, Krishen A, Asgharian A, Foster VJ et al. Seasonal affective disorder and its prevention by anticipatory treatment with bupropion XL. Biol Psychiatry 2005; 58(8): 658-667.

GlaxoSmithKline. A 7-month, multicenter, randomized, double-blind, placebo-controlled comparison of 150-300mg/day of extended-release bupropion hydrochloride (Wellbutrin XL) and placebo for the prevention of seasonal affective disorder in subjects with a history of seasonal affective disorder followed by an 8-week observational follow-up phase: clinical study report for study 100006 [unpublished]. 2004.

GlaxoSmithKline. A 7-month, multicenter, randomized, double-blind, placebo-controlled comparison of 150-300mg/day of extended-release bupropion hydrochloride (WELLBUTRIN XL) and placebo for the prevention of seasonal affective disorder in subjects with a history of seasonal affective disorder followed by an 8-week observational follow-up study: study no. WELL 100006 [online]. In: GlaxoSmithKline Clinical Study Register. 27.06.2005 [Access on: 25.08.2009]. URL: http://www.gsk-clinicalstudyregister.com/files/pdf/19890.pdf.

**Winokur 2003 [mirtazapine vs. fluoxetine]**

Winokur A, DeMartinis NA 3rd, McNally DP, Gary EM, Cormier JL, Gary KA. Comparative effects of mirtazapine and fluoxetine on sleep physiology measures in patients with major depression and insomnia. J Clin Psychiatry 2003; 64(10): 1224-1229.

**WXL101497 [venlafaxine vs. bupropion vs. placebo]**

GlaxoSmithKline. A multi-centre, randomised, double-blind, parallel-group, placebo- and active-controlled, flexible dose study evaluating the efficacy, safety and tolerability of extended-release bupropion hydrochloride (150 mg – 300 mg once daily), extended-release venlafaxine hydrochloride (75 mg – 150 mg once daily) and placebo in subjects with major depressive disorder: study WXL101497 [Online]. In: GlaxoSmithKline Clinical Trial Register. 06.02.2006 [Access on 22.01.2008]. URL: http://ctr.gsk.co.uk/Summary/bupropion/III_WXL101497.pdf.

GlaxoSmithKline. A multi-centre, randomised, double-blind, parallel-group, placebo- and active-controlled, flexible dose study evaluating the efficacy, safety and tolerability of extended-release bupropion hydrochloride (150 mg – 300 mg once daily), extended-release venlafaxine hydrochloride (75 mg – 150 mg once daily) and placebo in subjects with major depressive disorder: study WXL101497 [unpublished]. 2005.

**Zhang 2000 [venlafaxine vs. amitriptyline]**

Zhang JD, Feng RM, Ma C. A double blind comparison of venlafaxine and amitriptyline in treatment of depression. Guangzhou Medical Journal 2000; 31(3): 8-9.

**003-002 [mirtazapine vs. placebo]**

Claghorn JL, Johnstone EE, Studebaker SL, Ajeman SA. The effectiveness of 6-azamianserin (Org 3770) in depressed outpatients. Psychopharmacol Bull 1987; 23(1): 160-161.

Organon. A placebo-controlled study of Org 3770 in moderately depressed patients (protocol 003-002) [unpublished]. 1994.

Claghorn JL, Lesem MD. A double-blind placebo-controlled study of Org 3770 in depressed outpatients. J Affect Disord 1995; 34(3): 165-171.

**003-003 [mirtazapine vs. placebo]**

Organon. A placebo-controlled study of Org 3770 in moderately depressed outpatients: study no. 003-003 [unpublished]. 1993.

**003-008 [mirtazapine vs. placebo]**

Organon. A controlled dose range study of Org 3770 in outpatients with major depression: study no. 003-008 [unpublished]. 1994.

**003-020 [mirtazapine vs. placebo]**

Organon. A controlled study of Org 3770 in outpatients with major depression: study no. 003-020; clinical summary report [unpublished]. 1992.

**003-021 [mirtazapine vs. placebo]**

Organon. A controlled study of Org 3770 in out-patients with major depression: study no. 003-021 [unpublished]. 1992.

**003-022 [mirtazapine vs. placebo]**

Bremner JD. A double-blind comparison of Org 3770, amitriptyline, and placebo in major depression. J Clin Psychiatry 1995; 56(11): 519-525.

Organon. A controlled study of Org 3770 in outpatients with major depression: study no. 003-022; clinical summary report [unpublished]. 1989.

Bremner JD. Doppelblindvergleich von Mirtazapin, Amitriptylin und Plazebo bei 'Major Depression'. Nervenheilkunde 1996; 15(8): 533-540.

**003-023 [mirtazapine vs. trazodon vs. placebo]**

Halikas JA. Org 3770 (mirtazapine) versus trazodone: A placebo controlled trial in depressed elderly patients. Hum Psychopharmacol 1995; 10(Suppl 2): S125-S133.

Organon. A controlled study of Org 3770 in elderly outpatients with major depression: study no. 003-023 [unpublished]. 1992.

**003-024 [mirtazapine vs. placebo]**

Smith WT, Glaudin V, Panagides J, Gilvary E. Mirtazapine vs. amitriptyline vs. placebo in the treatment of major depressive disorder. Psychopharmacol Bull 1990; 26(2): 191-196.

Organon. A controlled study of Org 3770 in outpatients with major depression: study no. 003-024; clinical summary report [unpublished]. 1990.

**003-041 [mirtazapine vs. placebo]**

Thase ME, Nierenberg AA, Keller MB, Panagides J, Relapse Prevention Study G. Efficacy of mirtazapine for prevention of depressive relapse: a placebo-controlled double-blind trial of recently remitted high-risk patients. J Clin Psychiatry 2001; 62(10): 782-788.

Organon. A multicenter, placebo-controlled study of relapse prevention by long-term treatment with the recommended dose of Remeron in outpatients with major depressive episode: clinical report on protocol 003-041 [unpublished]. 1999.

Nierenberg AA, Quitkin FM, Kremer C, Keller MB, Thase ME. Placebo-controlled continuation treatment with mirtazapine: acute pattern of response predicts relapse. Neuropsychopharmacology 2004; 29(5): 1012-1018.

**003-042 [mirtazapine vs. placebo]**

Organon. An eight-week, multicenter, double-blind placebo-controlled fixed dose response study to define the antidepressant effectiveness and sedation properties of Remeron in outpatients with major depression: clinical trial report on protocol 003042 [unpublished]. 1999.

**003-048 [mirtazapine vs. fluoxetine vs. placebo]**

Organon. Multi-center, randomized, double-blind, fluoxetine and placebo-controlled study of the efficacy and safety of RemeronSolTab orally disintegrating tablets (mirtazapine ODT) in subjects with major depressive disorder: clinical trial report on protocol 003048 [unpublished]. 2003.

**003-900 [mirtazapine vs. sertaline]**

Organon. Multicenter, randomized, double-blind, sertraline-controlled study of the efficacy and safety of Remeron (mirtazapine) in subjects with major depressive disorder who failed on SSRI treatment due to lack of efficacy; clinical trial report on protocol 003-900 [unpublished]. 2001.

**003-901 [mirtazapine vs. paroxetine]**

Schatzberg AF, Kremer C, Rodrigues HE, Murphy GM Jr. Mirtazapine vs. Paroxetine Study G. Double-blind, randomized comparison of mirtazapine and paroxetine in elderly depressed patients. Am J Geriatr Psychiatry 2002; 10(5): 541-550.

Murphy GM Jr, Kremer C, Rodrigues HE, Schatzberg AF. Pharmacogenetics of antidepressant medication intolerance. Am J Psychiatry 2003; 160(10): 1830-1835.

Murphy GM, Kremer C, Rodrigues H, Schatzberg AF, Mitrazapine versus paroxetine Study G. The apolipoprotein E epsilon4 allele and antidepressant efficacy in cognitively intact elderly depressed patients. Biol Psychiatry 2003; 54(7): 665-673.

Murphy GM Jr, Hollander SB, Rodrigues HE, Kremer C, Schatzberg AF. Effects of the serotonin transporter gene promoter polymorphism on mirtazapine and paroxetine efficacy and adverse events in geriatric major depression. Arch Gen Psychiatry 2004; 61(11): 1163-1169.

Organon. Multicenter, randomized, double-blind, paroxetine-controlled study of the efficacy and safety of Remeron (mirtazapine) in subjects with major depressive disorder who are at least 65 years of age: clinical trial report on protocol 003-901 [unpublished]. 2001.

**013 [reboxetine vs. placebo]**

Versiani M, Mehilane L, Gaszner P, Arnaud-Castiglioni R. Reboxetine, a unique selective NRI, prevents relapse and recurrence in long-term treatment of major depressive disorder. J Clin Psychiatry 1999; 60(6): 400-406.

Pharmacia. Multicentre, multinational double-blind study of the activity and tolerability of reboxetine vs pIacebo in the continuation therapy of major depressive episodes (phase III): final report of study CTN013-FCE20124 [unpublished]. 1995.

**014 [reboxetine vs.fluoxetine vs. placebo]**

Andreoli V, Caillard V, Deo RS, Rybakowski JK, Versiani M. Reboxetine, a new noradrenaline selective antidepressant, is at least as effective as fluoxetine in the treatment of depression. J Clin Psychopharmacol 2002; 22(4): 393-399.

Dubini A, Bosc M, Polin V. Noradrenaline-selective versus serotonin-selective antidepressant therapy: differential effects on social functioning. J Psychopharmacol 1997; 11(4 Suppl): S17-S23.

Dubini A, Bosc M, Polin V. Do noradrenaline and serotonin differentially affect social motivation and behaviour? Eur Neuropsychopharmacol 1997; 7(Suppl 1): S49-S55

Pharmacia. Multicentre, multinational double-blind study of the activity and tolerability of reboxetine vs fluoxetine and placebo in patients suffering from major depressive episodes (phase III): results of patient self-evaluation assessment instrument; addendum to final report no 9550080 of study CTN014-FCE20124 [unpublished]. 1995.

**015 [reboxetine vs. imipramine vs. placebo]**

Pharmacia. Multicentre, multinatiotial double-blind study of the activity and tolerability of reboxetine vs imipramine and placebo in patients suffering from major depressive episodes (phase III): final report of study CTN015-FCE20124 [unpublished]. 1995.

**016 [reboxetine vs. fluoxetine]**

Massana J, Moller HJ, Burrows GD, Montenegro RM. Reboxetine: a double-blind comparison with fluoxetine in major depressive disorder. Int Clin Psychopharmacol 1999; 14(2): 73-80.

Pharmacia. Multicentre, multinational double-blind study of the activity and tolerability of reboxetine vs fluoxetine in patients suffering from major depressive episodes (phase III): final report of study CTN016-FCE20124 [unpublished]. 1995.

**017 [reboxetine vs. imipramine]**

Berzewski H, Van Moffaert M, Gagiano CA. Efficacy and tolerability of reboxetine compared with imipramine in a double-blind study in patients suffering from major depressive offsodes. Eur Neuropsychopharmacol 1997; 7(Suppl 1): S37-47.

Pharmacia. Multicentre, multinational double-blind study of the activity and tolerability of reboxetine vs imipramine in patients suffering from major depressive episodes (phase III): final report of study CTN017-FCE20124 [unpublished]. 1995.

**022 [reboxetine vs. dothiepin]**

Pharmacia. A phase IV study to compare the efficacy and tolerability of reboxetine versus dothiepin in subjects suffering from major depressive disorder in general practice: study no M2020/0022; final statistical report [unpublished]. 2000.

**032 [reboxetine vs. fluoxetine]**

Pharmacia. Reboxetine (PNU-155950E) vs fluoxetine in a double-blind study for the treatment of major depressive disorders in Taiwan: study report for M/2020/0032 [unpublished]. 2001.

**034 [reboxetine vs. placebo]**

Pharmacia & Upjohn. Reboxetine (PNU-155950E) vs. placebo in the treatment of major depressive disorder resistant to fluoxetine: final report of the trial M-2020-0034 [unpublished]. 2003.

**043 [reboxetine vs. citalopram]**

Langworth S, Bodlund O, Agren H. Efficacy and tolerability of reboxetine compared with citalopram: a double-blind study in patients with major depressive disorder. J Clin Psychopharmacol 2006; 26(2): 121-127.

Pharmacia. Efficacy and tolerability of reboxetine (PNU-155950E) compared to citalopram in a double-blind study in patients with major depressive disorder: study no. Z2020 0043; abbreviated study report; final version [unpublished]. 2003.

**045 [reboxetine vs. placebo]**

Pharmacia & Upjohn. Comparison of placebo and three fixed doses of reboxetine in a population of patients with major depression: a phase II, double-blind, randomized, parallel group, multicenter study of 3 fixed doses of reboxetine or placebo, given orally twice daily to adult patients with major depressive disorder; final report of the trial 95-CRBX-045 [unpublished]. 2001.

**046 [reboxetine vs. paroxetine vs. placebo]**

Pharmacia & Upjohn. Reboxetine, placebo, and paroxetine comparison in patients with major depressive disorder: a phase III, randomized, double-blind, placebo- and active-treatment-controlled, parallel-group, 8-week study of reboxetine, given orally twice daily to adult patients with major depressive disorder; final report of the study protocol M/2020/0046 [unpublished]. 2001.

**047 [reboxetine vs. paroxetine vs. placebo]**

Ferguson JM, Wesnes KA, Schwartz GE. Reboxetine versus paroxetine versus placebo: effects on cognitive functioning in depressed patients. Int Clin Psychopharmacol 2003; 18(1): 9-14.

Pharmacia & Upjohn. Reboxetine, placebo, and paroxetine comparison in patients with major depressive disorder: a phase III, randomized, double-blind, placebo- and active-treatment-controlled, parallel-group, 8-week study of reboxetine, given orally twice daily to adult patients with major depressive disorder: final report of the study protocol M/2020/0047 [unpublished]. 2001.

**049 [reboxetine vs. placebo]**

Pharmacia & Upjohn. Reboxetine (PNU-155950E) versus placebo in the treatment of major depressive disorders: final report of the trial protocol number 97-CRBX049 [unpublished]. 2001.

**050 [reboxetine vs. fluoxetine vs. placebo]**

Clayton AH, Zajecka J, Ferguson JM, Filipiak-Reisner JK, Brown MT, Schwartz GE. Lack of sexual dysfunction with the selective noradrenaline reuptake inhibitor reboxetine during treatment for major depressive disorder. Int Clin Psychopharmacol 2003; 18(3): 151-156.

Pharmacia & Upjohn. Reboxetine (PNU-155950E) versus placebo and fluoxetine in a controlled, randomized, double-blind, multicenter study of treatment in major depressive disorders: final report of the study protocol 97-CRBX-050 [unpublished]. 2001.

**052 [reboxetine vs. paroxetine]**

Baldwin D, Bridgman K, Buis C. Resolution of sexual dysfunction during double-blind treatment of major depression with reboxetine or paroxetine. J Psychopharmacol 2006; 20(1): 91-96.

Pharmacia & Upjohn. Reboxetine (PNU-155950E) vs paroxetine in a double-blind, multinational study of treatment in major depressive disorder: final report of the study 97-CRBX-052 [unpublished]. 2004.

**0600A-321-GMR-18105 [venlafaxine vs. maprotiline]**

Wyeth Pharma GmbH. A randomized, double-blind, parallel group comparison of venlafaxine and maprotiline capsules in outpatients with major depression: study 0600A-321-GMR-18105 [unpublished].

**0600-326-GMR-20004 [venlafaxine vs. clomipramine]**

Wyeth Pharma GmbH. A randomized double-blind parallel group comparison capsules in outpatients with major depression: study 0600A-326-GMR-20004 [unpublished].

**0600A1-343-US-GMR-25304 [venlafaxine vs. placebo]**

Wyeth Pharma GmbH. A double-blind, randomized, controlled evaluation of the effects of venlafaxine on blood pressure of patients being treated for major depression: study 0600A1-343-US-GMR-25304 [unpublished].

**600A1-347-FR-NE-CSR [venlafaxine vs. fluvoxamine]**

Wyeth Pharma GmbH. A double-blind, randomization study of the safety and efficacy of two regimes of venlafaxine compared with the regimen of fluvoxamine: study 0600A1-347-FRNE-CSR-40558 [unpublished].

**0600A1-349-NE-UK-CSR [venlafaxine vs. paroxetine]**

Wyeth Pharma GmbH. A double-blind, randomized 8-week, comparative study of the safety and efficacy of venlafaxine and paroxetine: study 0600A1-349-NE-UK-CSR-40241 [unpublished].

**0600A1_351_GE-CSR_43127 [venlafaxine vs. moclobemide]**

Wyeth Pharma GmbH. A double-blind randomized 6-week study of the safety and efficacy of Effexor (venlafaxine) compared with moclobemide in the treatment of major depression:study 0600A1-351-GE-CSR-43127 [unpublished].

**0600A1-372-US-GMR-32822 [venlafaxine vs. fluoxetine vs. placebo]**

Wyeth Pharma GmbH. A double-blind, placebo-controlled, parallel-group, comparative study of venlafaxine and fluoxetine in depressed outpatients to measure onset of clinical activity: study 0600A1-372-US-GMR-32822 [unpublished].

**0600B-367-EU GMR-25782 [venlafaxine vs. paroxetine vs. placebo]**

Wyeth Pharma GmbH. A randomized double-blind, placebo-controlled, fixed-dose study of efficacy and safety of venlafaxine extended release and paroxetine in depressed outpatients: study 0600B-367-EU GMR-25782 [unpublished].

**0600B-671-UK [venlafaxine vs. citalopram]**

Wyeth Pharma GmbH. A doube-blind, double-dummy, randomized study of the efficacy and safety of venlafaxine extended release (ER) compared to citalopram in depressed patients unresponsive to treatment with SSRI's: study 0600B-671-UK [unpublished].

**0600B1-384-US-EU-CA-CSR-41642 [venlafaxine vs. placebo]**

Wyeth Pharma GmbH. A double-blind, placebo-controlled, comparative study of extended release formulation of venlafaxine and imipramine on the time of onset of antidepressant response in patients with severe major depression: study 0600B1-384-US-EU-CA-CSR-41642 [unpublished].

**0600C1-217-US-CSR-45150 [venlafaxine vs. placebo]**

Wyeth Pharma GmbH. Double-blind placebo controlled study of venlafaxine ER and venlafaxine oros in outpatients with major depression: study 0600C1-217-US-CSR-45150 [unpublished].

**0600C1-402-US-CA-CSR-48579 [venlafaxine vs. sertaline vs. placebo]**

Wyeth Pharma GmbH. A double-blind, placebo-controlled, comparative efficacy study of venlafaxine and sertraline in producing remission in outpatients with major depressive disorder: study 0600C1-402-US-CA-CSR-48579 [unpublished].

**0600-332-US-GMR-21989 [venlafaxine vs. fluoxetine]**

Wyeth Pharma GmbH. A randomized double-blind comparison of venlafaxine and fluoxetine in outpatients with major depression: study 600A-332-US-GMR-21989 [unpublished].

**0600-428-IT-SDC-3993 [venlafaxine vs. paroxetine]**

Wyeth Pharma GmbH. A randomized double-blind comparision of venlafaxine XR and paroxetine in outpatients with moderate to severe major depression: study 0600-428-IT-SDC-3993 [unpublished].

**091 [reboxetine vs. placebo]**

Versiani M, Amin M, Chouinard G. Double-blind, placebo-controlled study with reboxetine in inpatients with severe major depressive disorder. J Clin Psychopharmacol 2000; 20(1): 28-34.

Versiani M. The selective noradrenaline re-uptake inhibitor reboxetine has an early onset of antidepressant action. Int J Psychiatry Clin Pract 2000; 4(4): 293-297.

Pharmacia. Phase II placebo-controlled clinical study with reboxetine in major depressions: study CTN:20124/ADE 091; clinical study report [unpublished]. 1993.

**22521 [mirtazapine vs. fluoxetine]**

Hong CJ, Hu WH, Chen CC, Hsiao CC, Tsai SJ, Ruwe FJL. A double-blind, randomized, group-comparative study of the tolerability and efficacy of 6 weeks' treatment with mirtazapine or fluoxetine in depressed Chinese patients. J Clin Psychiatry 2003; 64(8): 921-926.

Organon. A double-blind, randomized, fluoxetine-controlled, group-comparative study comparing the tolarablility and efficacy of six weeks treatment with Org 3770 and fluoxetine in depressed patients: clinical trial report on protocol 22521 (including 22521a, 22521b and 22521c) [unpublished]. 2001.

**22532 [mirtazapine vs. fluvoxamine]**

Organon. A double-blind, randomized, flexible dose, fluvoxamine-controlled, group-comparative trial, comparing the efficacy and safety of six weeks treatment with Org 3770 and fluvoxamine in patients suffering from major depressive disorder (according to DSM-IV): clinical trial report on protocol 22532 (Europe) / 9902 (Japan) [unpublished]. 2002.

**600A_303_US [venlafaxine vs. placebo]**

Wyeth Pharma GmbH. Randomized, double-blind comparison of venlafaxine (WY-45, 030), imipramine, and placebo capsules in outpatients with major depression: study 600A-303-US-303-EXT-GMR-20448 [unpublished].

**600A-654-AU [venlafaxine vs. fluoxetine]**

Wyeth Pharma GmbH. A double-blind, randomized 12-week study of the safety and efficacy of oral venlafaxine up to 75 mg bid compared with oral fluoxetine up to 20 mg bid in patients with moderate and severe major depression: study 600A-654-AU [unpublished].

**84023 [mirtazapine vs. placebo]**

Vartiainen H, Leinonen E. Double-blind study of mirtazapine and placebo in hospitalized patients with major depression. Eur Neuropsychopharmacol 1994; 4(2): 145-150.

Organon. A double-blind, placebo-controlled, flexible dose, efficacy and safety study with Org 3770 administered orally for a period of six weeks to hospitalized patients with major depressive episode (multicentre study): protocol no. 84023 [unpublished]. 1990.

**88013 [mirtazapine vs. amitriptyline]**

Hoyberg OJ, Maragakis B, Mullin J, Norum D, Stordall E, Ekdahl P et al. A double-blind multicentre comparison of mirtazapine and amitriptyline in elderly depressed patients. Acta Psychiatr Scand 1996; 93(3): 184-190.

Organon. A multicentre, double-blind, randomized, group-comparative study to evaluate the effects of six weeks treatment with Org 3770 and amitriptyline administered to elderly patients with major depressive disorder: study no. 88013 [unpublished]. 1992.

**9902 [mirtazapine vs. fluvoxamine]**

Organon. A double-blind, randomized, flexible dose, fluvoxamine-controlled, group-comparative trial, comparing the efficacy and safety of six weeks treatment with Org 3770 and fluvoxamine in patients suffering from major depressive disorder (according to DSM-IV): clinical trial report on protocol 22532 (Europe) / 9902 (Japan) [unpublished]. 2002.

Organon. A double-blind, randomized, flexible dose, fluvoxamine-controlled, group-comparative trial, comparing the efficacy and safety of six weeks treatment with Org 3770 and fluvoxamine in patients suffering from major depressive disorder (according to DSM-IV): clinical trial report on protocol 9902 [unpublished]. 2007.
